# ﻿Phylogeny and species delimitations in the economically, medically, and ecologically important genus *Samsoniella* (Cordycipitaceae, Hypocreales)

**DOI:** 10.3897/mycokeys.99.106474

**Published:** 2023-10-03

**Authors:** Yao Wang, Zhi-Qin Wang, Chinnapan Thanarut, Van-Minh Dao, Yuan-Bing Wang, Hong Yu

**Affiliations:** 1 Yunnan Herbal Laboratory, College of Ecology and Environmental Sciences, Yunnan University, Kunming 650504, China; 2 The International Joint Research Center for Sustainable Utilization of Cordyceps Bioresources in China and Southeast Asia, Yunnan University, Kunming 650504, China; 3 Faculty of Agricultural Production, Maejo University, Chiang Mai 50290, Thailand; 4 Institute of Regional Research and Development, Ministry of Science and Technology, Hanoi 100803, Vietnam

**Keywords:** *Isaria*-like fungi, multi-locus phylogeny, new species, species diversity

## Abstract

*Samsoniella* is a ubiquitous genus of cosmopolitan arthropod-pathogenic fungi in the family Cordycipitaceae. The fungi have economic, medicinal, and ecological importance. Prior taxonomic studies of these fungi relied predominantly on phylogenetic inferences from five loci, namely, the nuclear ribosomal small and large subunits (nr SSU and nr LSU), the 3’ portion of translation elongation factor 1 alpha (3P_*TEF*), and RNA polymerase II subunits 1 and 2 (*RPB1* and *RPB2*). Despite many new species being described, not all of the recognized species inside this group formed well-supported clades. Thus, the search for new markers appropriate for molecular phylogenetic analysis of *Samsoniella* remains a challenging problem. In our study, we selected the internal transcribed spacer regions of the rDNA (ITS rDNA) and seven gene regions, namely, 3P_*TEF*, the 5’ portion of translation elongation factor 1 alpha (5P_*TEF*), *RPB1*, *RPB2*, γ-actin (*ACT*), β-tubulin (*TUB*), and a gene encoding a minichromosome maintenance protein (*MCM7*), as candidate markers for species identification. Genetic divergence comparisons showed that the ITS, *RPB2*, *ACT*, and *TUB* sequences provided little valuable information with which to separate *Samsoniella* spp. In contrast, sequence data for 3P_*TEF*, 5P_*TEF*, *RPB1*, and *MCM7* provided good resolution of *Samsoniella* species. The phylogenetic tree inferred from combined data (5P_*TEF* + 3P_*TEF* + *RPB1* + *MCM7*) showed well-supported clades for *Samsoniella* and allowed for the delimitation of 26 species in this genus. The other two species (*S.formicae* and *S.lepidopterorum*) were not evaluated, as they had abundant missing data.

## ﻿Introduction

*Samsoniella* is a ubiquitous genus of cosmopolitan arthropod-pathogenic fungi containing several species with significant economic and medicinal value (Wang et al. 2022). *Samsoniellahepiali* is a well-known edible and medicinal fungus that is widely distributed in China and Argentina (Chen et al. 2021). Several studies have shown that *S.hepiali* possesses various pharmacological properties, including anti-cancer, analgesic, and proapoptotic activities (Dai et al. 1989; Jiang et al. 2010; Thakur et al. 2011; Wang et al. 2016). The fungus has been frequently used in China for the treatment of cardiovascular disease, respiratory conditions, hyposexuality, hyperglycemia, and renal disorders, as it has immunomodulatory effects that result in clearing of the lungs, lowering blood glucose, and reinforcing kidney function (Lou et al. 1986; Huang et al. 1988; Wang and Huang 1988; Dai et al. 1989; Zou and Huang 1993; Xiang et al. 2006). To date, more than 260 healthcare products in the world market have been developed with *S.hepiali* as a raw material, creating an economic value of approximately RMB10 billion ($1.46 billion) per year (Wang et al. 2020a). *Samsoniellafarinospora* also has potential to be further developed into future healthcare products; this species and *S.hepiali* have a close genetic relationship and similar pharmacological activities (Wang et al. 2022). The arthropod-pathogenic fungi *Samsoniella* spp. are widely distributed and infest diverse hosts, and some species have been considered as potential biocontrol agents against pest insects (Wang et al. 2022).

**Figure 1.**
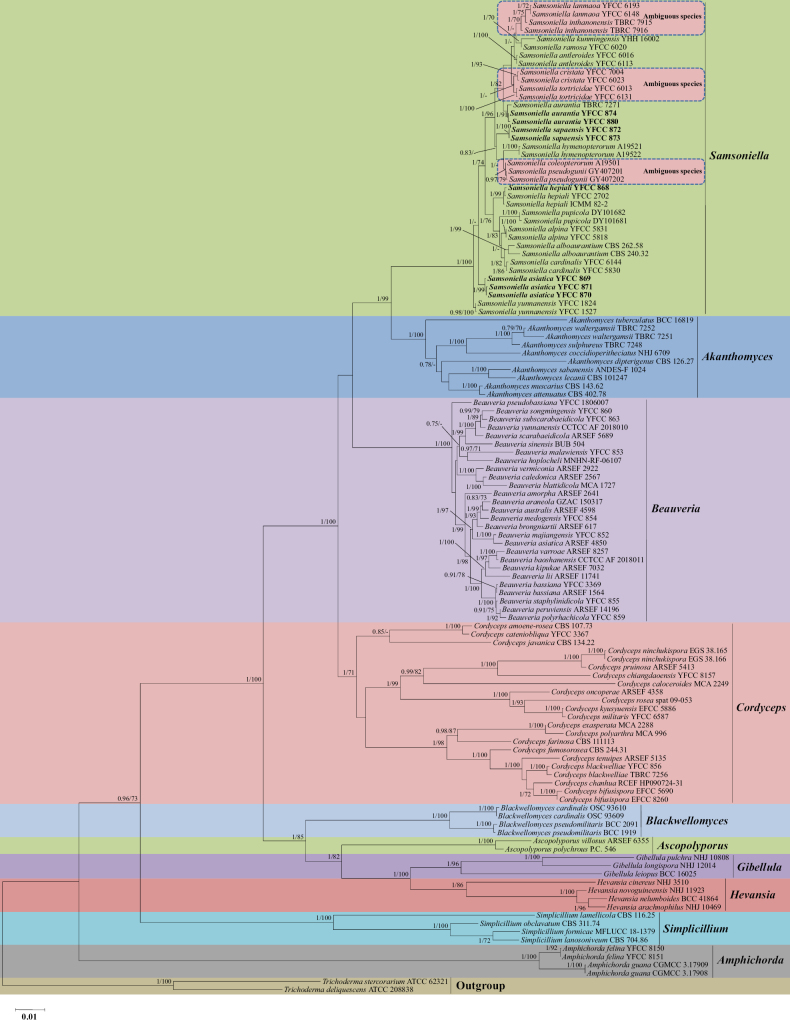
Phylogenetic relationships among the genus *Samsoniella* and its allies in Cordycipitaceae based on Bayesian inference (BI) and maximum likelihood (ML) analyses of a five-locus (nr SSU, nr LSU, 3P_*TEF*, *RPB1*, and *RPB2*) dataset. No significant differences in topology are observed between BI and ML (RAxML) phylogenies. Statistical support values (≥ 0.7/70%) are shown at the nodes for BI posterior probabilities/ML bootstrap support. Materials in bold type are those analyzed in this study.

The genus *Samsoniella* was established on the basis of three species with orange cylindrical to clavate stromata, superficial perithecia, and orange conidiophores with *Isaria*-like phialides and white to cream conidia: the type species *S.inthanonensis* and two other species, *S.alboaurantia* and *S.aurantia* (Mongkolsamrit et al. 2018). However, it is difficult to identify individual species of *Samsoniella* using only morphological characteristics (Wang et al. 2022). Given the problems with species delimitation in *Samsoniella* using morphology, molecular data are essential to establish robust species boundaries. Mongkolsamrit et al. (2018) segregated the above three *Isaria*-like species from the *Akanthomyces* group based on the nuclear ribosomal small and large subunits (nr SSU and nr LSU) and three nuclear genes encoding elongation factor 1 alpha (3P_*TEF*, the 3’ portion of translation elongation factor 1 alpha), RNA polymerase II largest subunit (RPB1), and RNA polymerase II second largest subunit (*RPB2*). Subsequently, more than 15 new species and new combinations were erected using combined analysis of the five-locus sequence data (Wang et al. 2020a; Wang et al. 2022). However, recent phylogenetic analyses together with our five-gene phylogeny of the family Cordycipitaceae (Fig. 1) showed that: (1) not all of the recognized species in this group formed well-supported clades; (2) the clade composed of *S.inthanonensis* / *S.lanmaoa*, *S.cristata* / *S.tortricidae*, and *S.coleopterorum* / *S.pseudogunii* showed ambiguous positions; and (3) the genetic distances of *Samsoniella* species for the combined five-gene sequences were significantly lower than for species of related genera within Cordycipitaceae.

In the current study, we analyzed species of the recently circumscribed genus *Samsoniella*, based on morphological observations and phylogenetic inference. Moreover, we selected the internal transcribed spacer regions of the rDNA (ITS rDNA) and six protein-coding genes (elongation factor EF-1α (3P_*TEF* and 5P_*TEF*, the 5’ portion of translation elongation factor 1 alpha), RNA polymerases (*RPB1* and *RPB2*), γ-actin (*ACT*), β-tubulin (TUB), and a gene encoding a minichromosome maintenance protein (*MCM7*)) for evaluation as taxonomic candidate markers for phylogenetic inference; these have been commonly used in fungal species identification and in phylogenetic reconstructions of fungi (AFTOL, http://aftol.org/data.php). Finally, nucleotide sequences derived from four markers, namely, 3P_*TEF*, 5P_*TEF*, *RPB1*, and *MCM7*, were used to examine phylogenetic relationships and assess species boundaries within the genus.

## ﻿Materials and methods

### ﻿Specimen collection and fungus isolation

Fungus-infected insect specimens were collected from seven locations in 2016 and 2020, including three different locations within Yunnan Province, China, two locations within Lao Cai Province, Vietnam, one location within Oudomxay Province, Laos, and one location in Chiang Mai, Thailand. Teleomorph specimens were collected by carefully unearthing their hosts with a scoop and placing the samples in sterile bags. Conidia developing on insect cadavers were transplanted onto plates of potato dextrose agar (PDA; potato 200 g/L, dextrose 20 g/L, agar 20 g/L) and cultured at 25 °C. Colonies of the isolated filamentous fungi appearing in the culture were transferred onto fresh PDA media. The purified fungal strain was transferred to PDA slants and cultured at 25 °C until hyphae spread across the entire slope. Emerging fungal spores were washed with sterile physiological saline and made into a spore suspension of 1 × 10^3^ cells/mL. To obtain monospore cultures, a portion of the spore suspension was placed on PDA using a sterile micropipette, and then incubated at 25 °C. Teleomorph specimens were rinsed with tap water, washed with sterile distilled water, and then dried on sterile filter paper. A mass of ascospores and asci was removed from perithecia with a fine needle and placed in a drop of sterile water that was stirred with a different needle to distribute the elements on the slide. A portion of the drop containing ascospores was placed on PDA using a sterile micropipette, and then incubated at 25 °C. The purified fungal strains were maintained in a culture room at 25 °C or transferred to PDA slants and stored at 4 °C. Voucher specimens and the corresponding isolated strains were deposited in the Yunnan Herbal Herbarium (YHH) and the Yunnan Fungal Culture Collection (YFCC), respectively, of Yunnan University, Kunming, China.

### ﻿Morphological observations

Macro-morphological characteristics, including the host, geographical location, color and shape of the stromata, and perithecial orientation (superficial, immersed, or semi-immersed; ordinal or oblique) were examined under a dissecting microscope (SZ61, Olympus Corporation, Tokyo, Japan). For morphological evaluation, microscope slides were prepared by placing mycelia from the cultures on PDA blocks (5 mm in diameter) and then overlaid with a coverslip. Medan dye solution was used to stain asci and ascospores. Other structures were mounted in water. The sizes and shapes of the microcharacteristics (e.g., asci, ascospores, conidiogenous cells, and conidia) were determined using a light microscope (CX40, Olympus Corporation, Tokyo, Japan) and a scanning electron microscope (Quanta 200 FEG, FEI Company, Hillsboro, USA). Individual length and width measurements were taken for 30–100 replicates, including the absolute minima and maxima.

### ﻿DNA extraction and PCR amplification

Specimens and live axenic cultures were prepared for DNA extraction. Genomic DNA was extracted using a Genomic DNA Purification Kit (Qiagen GmbH, Hilden, Germany) according to the manufacturer’s protocol. The phylogenetic positions of unknown *Samsoniella* isolates were evaluated with phylogenetic inferences based on five genes, namely, nr SSU, nr LSU, 3P_*TEF*, *RPB1*, and *RPB2*. The primer pair nrSSU-CoF and nrSSU-CoR (Wang et al. 2015) was used to amplify nr SSU; the primer pair LR5 and LR0R (Vilgalys and Hester 1990; Rehner and Samuels 1994) was used to amplify nr LSU, and the primer pair 983F and 2218R (Rehner and Buckley 2005) was used to amplify 3P_*TEF*. For amplification of the *RPB1* and *RPB2* genes, PCR primer pairs RPB1-5’F / RPB1-5’R and RPB2-5’F / RPB2-5’R (Bischoff et al. 2006; Sung et al. 2007) were employed. In total, seven DNA regions were evaluated as potential DNA barcodes for establishing species boundaries in *Samsoniella* because of their wide usage in phylogenetic studies of the sac-fungi. The markers included a fragment of the ITS region using primers ITS5 and ITS4 (White et al. 1990), a fragment of the 5P_*TEF* region using primers EF1T and EF2T (Rehner and Buckley 2005; Bischoff et al. 2006), a fragment of the 3P_*TEF*, *RPB1* and *RPB2* region with the same primer sets mentioned previously, a fragment of the *ACT* region using primers Act-1 and Act-4R (Voigt and Wöstemeyer 2000), a fragment of the *TUB* region using primers Bt2a and Bt1b (Glass and Donaldson 1995), and a fragment of the *MCM7* region using primers Mcm7-709for and Mcm7-1348rev (Schmitt et al. 2009). All of the PCR reactions were performed in a final volume of 50 μL containing 25 μL 2 × Taq PCR Master Mix (Tiangen Biotech Co., Ltd., China), 0.5 μL of each primer (10 μM), 1 μL of genomic DNA, and 23 μL of RNase-Free water. PCR products were sequenced by Beijing Sinogenomax Co., Ltd., China.

### ﻿Phylogenetic analyses

Amplified fragments were sequenced in both directions using the same primer pairs used for amplification. All retrieved sequences from GenBank were combined with those generated in our study. The taxonomic information and GenBank accession numbers are provided in Suppl. material 1 and Table 1. Sequences were aligned using MAFFT v.7 (http://mafft.cbrc.jp/alignment/server/). The aligned sequences were then manually corrected where necessary. Following alignment, the sequences of the five genes, namely, nr SSU, nr LSU, 3P_*TEF*, *RPB1*, and *RPB2*, were concatenated. Conflicts among the five genes were resolved using PAUP* 4.0b10 (Swofford 2002). The results showed that the phylogenetic signals for the five loci were congruent (*P* = 0.02). The best-fitting substitution model for these 11 partitions was calculated by using jModelTest version 2.1.4 (Darriba et al. 2012). The model GTR+G+I was used for partitions of nr SSU, nr LSU, 3P_*TEF*_pos1, 3P_*TEF*_pos2, 3P_*TEF*_pos3, *RPB1*_pos3, and *RPB2*_pos3, and the model GTR+I was used for partitions of *RPB1*_pos1, *RPB1*_pos2, *RPB2*_pos1, and *RPB2*_pos2. Bayesian posterior probabilities (BP) were estimated with the same partition parameters conducted in MrBayes v3.2.7a (Ronquist et al. 2012). Four Markov Chain Monte Carlo chains were run, each beginning with a random tree and sampling, one tree every 100 generations of 2,000,000 generations, and the first 25% of samples were discarded as burn-in. Maximum likelihood (ML) phylogenetic analyses were conducted in RAxML 7.0.3 (Stamatakis et al. 2008) with the recommended partition parameters, and 1000 rapid bootstrap replicates were performed on the dataset.

**Table 1.** Specimen information and GenBank accession numbers for sequences used in this study. Boldface: data generated in this study.

Taxon	Voucher information	GenBank accession number							
		3P*_TEF*	5P*_TEF*	*RPB1*	*RPB2*	*ACT*	*TUB*	*MCM7*	ITS
*Samsoniellaalboaurantium*	CBS 240.32	JF416019		JN049895	JF415999				AY624178
*Samsoniellaalboaurantium*	CBS 262.58^T^	MF416497		MF416654	MF416448				AY624179
*Samsoniellaalpina*	YFCC 5818^T^	MN576979	**OQ506160**	MN576869	MN576923	**OQ506202**	**OQ506254**	**OQ506229**	**OQ476469**
*Samsoniellaalpina*	YFCC 5831	MN576980	**OQ506161**	MN576870	MN576924	**OQ506203**	**OQ506255**	**OQ506230**	**OQ476470**
*Samsoniellaantleroides*	YFCC 6016^T^	MN576973	**OQ506162**	MN576863	MN576917	**OQ506204**	**OQ506256**	**OQ506231**	**OQ476471**
*Samsoniellaantleroides*	YFCC 6113	MN576974	**OQ506163**	MN576864	MN576918	**OQ506205**	**OQ506257**	**OQ506232**	**OQ476472**
***Samsoniellaasiatica***	**YFCC 869^T^**	**OQ506153**	**OQ506164**	**OQ506195**	**OQ506187**	**OQ506206**	**OQ506258**	**OQ506233**	**OQ476473**
***Samsoniellaasiatica***	**YFCC 870**	**OQ506154**	**OQ506165**	**OQ506196**	**OQ506188**	**OQ506207**	**OQ506259**	**OQ506234**	**OQ476474**
***Samsoniellaasiatica***	**YFCC 871**	**OQ506155**	**OQ506166**	**OQ506197**	**OQ506189**	**OQ506208**	**OQ506260**	**OQ506235**	**OQ476475**
*Samsoniellaaurantia*	TBRC 7271^T^	MF140846		MF140791	MF140818				MF140764
***Samsoniellaaurantia***	**YFCC 874**	**OQ506157**	**OQ506167**	**OQ506199**	**OQ506191**	**OQ506209**	**OQ506261**	**OQ506236**	**OQ476476**
***Samsoniellaaurantia***	**YFCC 880**	**OQ506156**	**OQ506168**	**OQ506198**	**OQ506190**	**OQ506210**	**OQ506262**	**OQ506237**	**OQ476477**
*Samsoniellacardinalis*	YFCC 5830	MN576958	**OQ506169**	MN576848	MN576902	**OQ506211**	**OQ506263**	**OQ506238**	**OQ476478**
*Samsoniellacardinalis*	YFCC 6144^T^	MN576956	**OQ506170**	MN576846	MN576900	**OQ506212**	**OQ506264**	**OQ506239**	**OQ476479**
*Samsoniellacoccinellidicola*	YFCC 8772^T^	ON676514		ON676502	ON568685				
*Samsoniellacoccinellidicola*	YFCC 8773	ON676515		ON676503	ON568686				
*Samsoniellacoleopterorum*	A19501^T^	MN101586		MT642600	MN101585				MT626376
*Samsoniellacristata*	YFCC 6023	MN576962	**OQ506171**	MN576852	MN576906	**OQ506213**	**OQ506265**	**OQ506240**	**OQ476480**
*Samsoniellacristata*	YFCC 7004^T^	MN576963	**OQ506172**	MN576853	MN576907	**OQ506214**	**OQ506266**	**OQ506241**	**OQ476481**
*Samsoniellaerucae*	KY11121^T^	ON525425							ON502828
*Samsoniellaerucae*	KY11122	ON525427							ON502847
*Samsoniellafarinospora*	YFCC 8774^T^	ON676516		ON676504	ON568687				
*Samsoniellafarinospora*	YFCC 9051	ON676517		ON676505	ON568688				
*Samsoniellaguizhouensis*	KY11161^T^	ON525429							ON502823
*Samsoniellaguizhouensis*	KY11162	ON525431							ON502845
*Samsoniellahaniana*	YFCC 8769^T^	ON676518		ON676506	ON568689				
*Samsoniellahaniana*	YFCC 8771	ON676520		ON676508	ON568691				
*Samsoniellahepiali*	ICMM 82-2^T^	MN576964	**OQ506173**	MN576854	MN576908	**OQ506215**	**OQ506267**	**OQ506242**	**OQ476482**
***Samsoniellahepiali***	**YFCC 868**	**OQ506158**	**OQ506175**	**OQ506200**	**OQ506192**	**OQ506217**	**OQ506269**	**OQ506244**	**OQ476484**
*Samsoniellahepiali*	YFCC 2702	MN576966	**OQ506174**	MN576856	MN576910	**OQ506216**	**OQ506268**	**OQ506243**	**OQ476483**
*Samsoniellahymenopterorum*	A19521	MN101588		MT642603	MT642604				MN128224
*Samsoniellahymenopterorum*	A19522^T^	MN101591		MN101589	MN101590				MN128081
*Samsoniellainthanonensis*	TBRC 7915^T^	MF140849		MF140790	MF140815				MF140761
*Samsoniellakunmingensis*	YHH 16002^T^	MN576972		MN576862	MN576916	**OQ506218**	**OQ506270**		
*Samsoniellalanmaoa*	YFCC 6148^T^	MN576959	**OQ506176**	MN576849	MN576903	**OQ506219**	**OQ506271**	**OQ506245**	**OQ476485**
*Samsoniellalanmaoa*	YFCC 6193	MN576960	**OQ506177**	MN576850	MN576904	**OQ506220**	**OQ506272**	**OQ506246**	**OQ476486**
*Samsoniellaneopupicola*	KY11321^T^	ON525433							ON502843
*Samsoniellaneopupicola*	KY11322	ON525435							ON502834
*Samsoniellapseudogunii*	GY407201^T^	MZ855233			MZ855239				MZ827470
*Samsoniellapseudogunii*	GY407202	MZ855234			MZ855240				MZ831863
*Samsoniellapseudotortricidae*	YFCC 9052^T^	ON676521		ON676509	ON568692				
*Samsoniellapseudotortricidae*	YFCC 9053	ON676522		ON676510	ON568693				
*Samsoniellapupicola*	DY101681^T^	MZ855231			MZ855237				MZ827085
*Samsoniellapupicola*	DY101682	MZ855232			MZ855238				MZ827008
*Samsoniellaramosa*	YFCC 6020^T^	MN576975	**OQ506178**	MN576865	MN576919	**OQ506221**	**OQ506273**		**OQ476487**
***Samsoniellasapaensis***	**YFCC 872**	**OQ506151**	**OQ506179**	**OQ506193**	**OQ506185**	**OQ506222**	**OQ506274**	**OQ506247**	**OQ476488**
***Samsoniellasapaensis***	**YFCC 873^T^**	**OQ506152**	**OQ506180**	**OQ506194**	**OQ506186**	**OQ506223**	**OQ506275**	**OQ506248**	**OQ476489**
*Samsoniellasinensis*	YFCC 8766^T^	ON676523		ON676511	ON568694				
*Samsoniellasinensis*	YFCC 8767	ON676524		ON676512	ON568695				
*Samsoniellatiankengensis*	KY11741^T^	ON525437							ON502840
*Samsoniellatiankengensis*	KY11742	ON525439							ON502849
*Samsoniellatortricidae*	YFCC 6131^T^	MN576976	**OQ506181**	MN576866	MN576920	**OQ506224**	**OQ506276**	**OQ506249**	**OQ476490**
*Samsoniellatortricidae*	YFCC 6142	MN576978	**OQ506182**	MN576868	MN576922	**OQ506225**	**OQ506277**	**OQ506250**	**OQ476491**
*Samsoniellayunnanensis*	YFCC 1527^T^	MN576982	**OQ506183**	MN576872	MN576926	**OQ506226**	**OQ506278**	**OQ506251**	**OQ476492**
*Samsoniellayunnanensis*	YFCC 1824	MN576983	**OQ506184**	MN576873	MN576927	**OQ506227**	**OQ506279**	**OQ506252**	**OQ476493**
*Akanthomyceswaltergamsii*	YFCC 883	**OQ506159**		**OQ506201**		**OQ506228**	**OQ506280**	**OQ506253**	**OQ476494**

Boldface: data generated in this study. ^T^ex-type strain.

We applied a (phylo-) genetic distance matrix calculation for the candidate markers, namely, ITS, 3P_*TEF*, 5P_*TEF*, *RPB1*, *RPB2*, *ACT*, *TUB*, and *MCM7*, to assess species boundaries of 11 *Samsoniella* spp. (Suppl. material 2) because their sequence data for the eight loci were complete. The pairwise genetic distances of the 11 *Samsoniella* lineages were measured based on the Kimura two-parameter model using MEGA v6.06 software (Tamura et al. 2013). Only candidate markers with the mean threshold criteria (p-distances) > 0.01 were used to examine phylogenetic relationships between *Samsoniella* spp. and to assess species boundaries within the genus. As a result, four markers, namely, 3P_*TEF*, 5P_*TEF*, *RPB1*, and *MCM7*, were singled out. Phylogenetic analyses were based on the combined four-locus (5P_*TEF* + 3P_*TEF* + *RPB1* + *MCM7*) sequences. The best-fitting nucleotide substitution model was determined using PartitionFinder V1.1.1 (Lanfear et al. 2012), resulting in three partitions (5P_*TEF* + 3P_*TEF*, *RPB1*, and *MCM7*). The following models were implemented in the Bayesian phylogenetic analyses: GTR + I + G for 5P_*TEF* + 3P_*TEF* and GTR + I for partitions of *RPB1* and *MCM7*. The BI analysis was run on MrBayes v3.2.7a for five million generations. GTR + FO + G was selected as the optimal model for ML analysis, and 1000 rapid bootstrap replicates were performed on the dataset. ML phylogenetic analyses were conducted in RAxML 7.0.3 (Stamatakis et al. 2008). Additional ML analyses were performed using IQ-TREE v. 2.1.3 with ultrafast bootstrapping for the estimation of branch support (Minh et al. 2020). Further, ML analysis (RAxML) was applied to single-locus genealogies for 5P_*TEF*, 3P_*TEF*, *RPB1*, and *MCM7*.

### ﻿Identification of host insects

The host insects of *Samsoniella* spp. were identified on the basis of morphological characteristics and further identified using molecular analyses based on the mitochondrial cytochrome oxidase I gene (COX1) and mitochondrial cytochrome b gene (CYTB). Genomic DNA was extracted from the head and leg areas of the cadavers of the host insects using the CTAB method (Liu et al. 2001). The COX1 and CYTB loci were amplified using primer pair Hep-cox1F / Hep-cox1R and primer pair Hep-cytbF / Hep-cytbR, respectively (Simon et al. 1994). Sequences were analyzed using MEGA v6.06 software and run through Standard Nucleotide BLAST (Genbank, NCBI nucleotide database) to assess similarity with reported insect sequences.

## ﻿Results

### ﻿Sequencing and phylogenetic analyses

The 11 DNA loci were readily amplified and sequenced, and there was a fairly high success rate in this study. Phylogenetic analyses based on the combined five-gene (nr SSU + nr LSU + 3P_*TEF* + *RPB1* + *RPB2*) sequences from 120 fungal taxa confirmed the presence and positions of *Samsoniella* and related genera within Cordycipitaceae. The concatenated five-gene dataset consisted of 4994 bp (nr SSU, 1134 bp; nr LSU, 901 bp; 3P_*TEF*, 1044 bp; *RPB1*, 759 bp; *RPB2*, 1156 bp). Eleven well-supported clades were recognized based on both BI and ML analyses of the combined dataset from Cordycipitaceae and *Trichoderma*, corresponding to the genera *Akanthomyces*, *Amphichorda*, *Ascopolyporus*, *Beauveria*, *Blackwellomyces*, *Cordyceps*, *Gibellula*, *Hevansia*, *Samsoniella*, *Simplicillium*, and *Trichoderma* as the outgroup (Fig. 1). A collection of eight isolates of unknown identity were shown to resolve in *Samsoniella* and to likely represent two known species and two new species of *Samsoniella*. Sequenced strains resolving in aforementioned distinct clades are thus proposed to represent the hereby newly erected species *S.asiatica* and *S.sapaensis*. The phylogenetic analyses suggested the existence of distinct species in the *Samsoniella* clade that we accordingly propose as new species: *S.asiatica* and *S.sapaensis*. These results also showed some ambiguous positions, including those of *S.inthanonensis* / *S.lanmaoa*, *S.cristata* / *S.tortricidae*, and *S.coleopterorum* / *S.pseudogunii* (Fig. 1). Although morphological observations revealed some differences in the characteristics between the three pairs of ambiguous species (Mongkolsamrit et al. 2018; Wang et al. 2020a; Chen et al. 2021), they were practically indistinguishable in the phylogeny based on the sequences of the five genes. This suggests that their status as distinct species is subjective and warrants further critical analyses, including the necessity for more DNA molecular markers.

The genetic divergence comparisons showed that: (1) the mean thresholds (p-distances) of ITS, *RPB2*, *ACT*, and *TUB* were lower than 0.01, indicating that neither were qualified as DNA markers; (2) the mean thresholds (p-distances) of 3P_*TEF*, 5P_*TEF*, *RPB1*, and *MCM7* were > 0.01, and (3) the highest number of species was delimited in the genetic distance analysis for the 3P_*TEF* sequence data, followed by 5P*_TEF*, *MCM7*, and *RPB1* sequences (Suppl. material 2).

**Figure 2.**
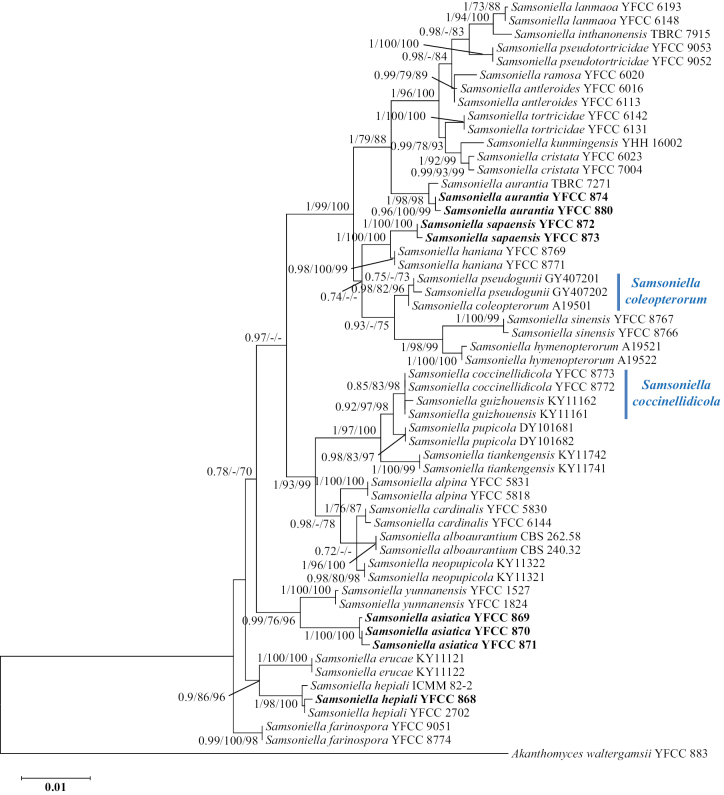
Phylogenetic tree of *Samsoniella* based on Bayesian inference and Maximum Likelihood analyses of a 4-locus (5P_*TEF*, 3P_*TEF*, *RPB1* and *MCM7*) dataset. No significant differences in topology are observed between BI and ML (IQ-TREE) phylogenies. Numbers at the branches indicate support values (BI-PP/RAxML-BS/IQ-TREE-BS) above 0.7/70%/70%. Isolates in bold type are those analyzed in this study.

The analyzed data matrix used to construct the phylogeny of *Samsoniella* species included sequences from 56 fungal taxa (Table 1). The final dataset consisted of 3130 bp of sequence data, including gaps (5P_*TEF*, 743 bp; 3P_*TEF*, 1023 bp; *RPB1*, 735 bp; and *MCM7*, 629 bp). Both BI and ML analyses produced trees with similar topologies that resolved most of the *Samsoniella* lineages in separate terminal branches (Fig. 2). The conservative results from phylogenetic analyses suggested that *Samsoniella* is composed of 26 species, namely, *S.alboaurantium*, *S.alpina*, *S.antleroides*, *S.asiatica*, *S.aurantia*, *S.cardinalis*, *S.coccinellidicola* (= *S.guizhouensis*), *S.coleopterorum* (= *S.pseudogunii*), *S.cristata*, *S.erucae*, *S.farinospora*, *S.haniana*, *S.hepiali*, *S.hymenopterorum*, *S.inthanonensis*, *S.kunmingensis*, *S.lanmaoa*, *S.neopupicola*, *S.pseudotortricidae*, *S.pupicola*, *S.ramosa*, *S.sapaensis*, *S.sinensis*, *S.tiankengensis*, *S.tortricidae*, and *S.yunnanensis*. The trees based on the combined five-locus sequences (nr SSU + nr LSU + 3P_*TEF* + *RPB1* + *RPB2*) and the combined four-locus (5P_*TEF* + 3P_*TEF* + *RPB1* + *MCM7*) sequences showed topological differences, especially in the clades composed of *S.inthanonensis* / *S.lanmaoa* and *S.cristata* / *S.tortricidae* (Figs 1, 2). However, the latter resolved these clades, suggesting that they should be regarded as different species.

The tree topologies for the individual loci (5P_*TEF*, 3P_*TEF*, *RPB1*, and *MCM7*) did not show congruence (Suppl. material 3). However, in all of the analyses, the newly discovered species *S.asiatica* had a close genetic relationship with *S.yunnanensis*. The *RPB1* gene was unable to distinguish the two species (Suppl. material 3: fig. S3). However, they were regarded as different species with strong support from 5P_*TEF*, 3P_*TEF* and *MCM7* (Suppl. material 3: figs S1, S2, S4). Phylogenetic analyses based on the combined data revealed that the distinctive species *S.sapaensis* was closely related to *S.haniana*, which is in agreement with the results obtained for 3P_*TEF* and *RPB1*.

### ﻿Taxonomy

Based on the results of the phylogenetic analyses and the morphological data, we add two new descriptions to the record of two known species and propose to erect two new species of *Samsoniella*.

#### ﻿ Samsoniella asiatica

Taxon classificationFungiHypocrealesCordycipitaceae

H. Yu bis, Y. Wang & Z.Q. Wang sp. nov.

265364D3-A77C-50DE-8E38-D67502CBA6F3

 848022

Fig. 3

##### Etymology.

Named after Asia (China, Vietnam and Laos), where the species was originally collected.

**Figure 3.**
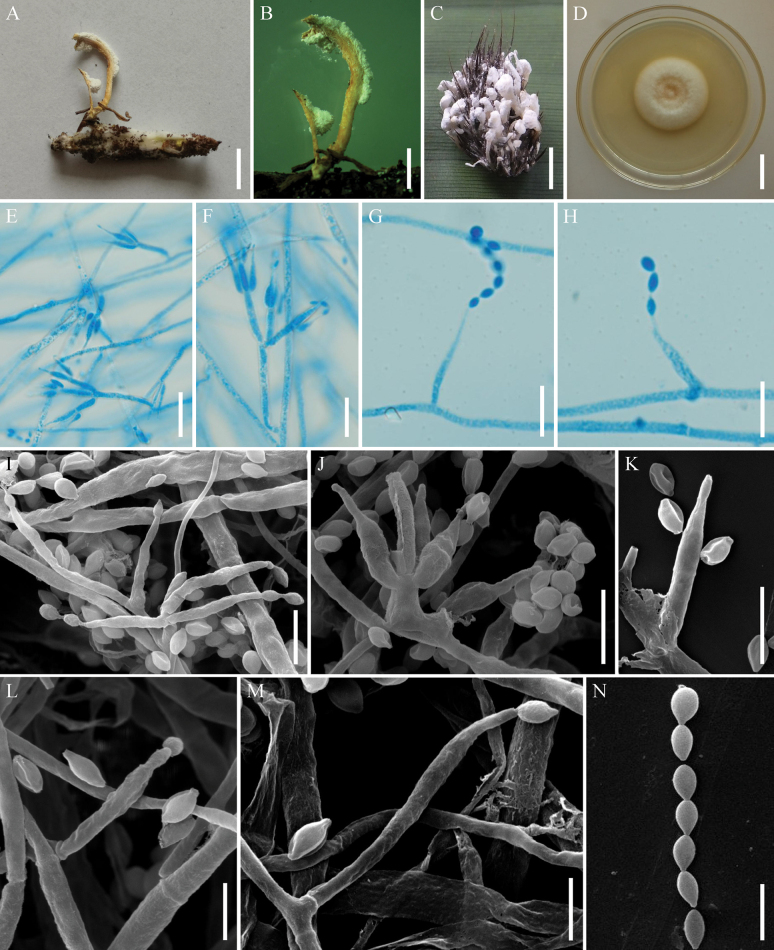
*Samsoniellaasiatica***A** fungus-infected lepidopteran pupa **B** stipes producing a mass of conidia at the apex **C** synnemata of fungus arising from lepidopteran larva **D** colony as obversed on PDA medium **E–M** conidiophores, phialides and conidia on PDA**N** conidia on PDA. Scale bars: 10 mm (**A, C**); 5 mm (**B**); 20 mm (**D**); 15 μm (**E**); 10 μm (**F, G, H**); 5 μm (**I, J, K, N**); 3 μm (**L, M**).

##### Type.

China, Yunnan Province, Yuanyang County, Xinjie Town, Duoyishu Village (23°4′50″N, 102°48′34″E, 1866 m above sea level), on a pupa of Lepidoptera in a dead twig, 10 December 2021, Yao Wang (holotype: YHH 869; ex-type living culture: YFCC 869).

##### Description.

**Teleomorph**: Undetermined. **Anamorph**: Synnemata arising from lepidopteran insects. Synnemata erect, flexuous, white or pale orange, 4–26 × 0.4–1.5 mm. Stipes cylindrical, producing a mass of conidia at the branches of synnemata, powdery and floccose. Colonies on PDA moderately fast-growing, 41–45 mm diameter in 14 days at 25 °C, white, cottony, generating several concentric rings at the centrum, sporulating abundantly, reverse white to pale yellow. Hyphae smooth-walled, branched, septate, hyaline, 1.3–2.0 µm wide. Conidiophores smooth-walled, cylindrical, solitary or verticillate, 4.6–10.3 × 0.8–1.9 µm. Phialides on conidiophores verticillate, usually in whorls of two to four, or solitary on hyphae, 2.7–8.6 µm long, basal portion cylindrical to narrowly lageniform, tapering gradually or abruptly toward the apex, from 0.7–1.7 µm wide (base) to 0.6–1.1 µm wide (apex). Conidia smooth and hyaline, fusiform or oval, one-celled, 1.1–1.8 × 0.8–1.2 µm, often in chains. Size and shape of phialides and conidia similar in culture and on natural substratum.

##### Distribution.

Yunnan Province, China; Lao Cai Province, Vietnam; Oudomxay Province, Laos.

##### Additional materials examined.

Vietnam, Lao Cai Province, Sa Pa District, Hoang Lien Mountains (22°21′4″N, 103°46′29″E, 1931 m above sea level), on a larva of Noctuidae buried in soil, 31 October 2016, collected by Hong Yu (YHH 871; living culture: YFCC 871); Laos, Oudomxay Province, Muang Xay County, Nagang Village (20°42′51″N, 102°5′44″E, 698 m above sea level), on a larva of *Spilosoma*, 29 July 2019, Yao Wang (YHH 870; living culture: YFCC 870).

##### Commentary.

Morphologically, *S.asiatica* resembles the phylogenetically closely related sister species *S.yunnanensis* in producing orange to pink stipes, a mass of conidia toward the apex synnemata and *Isaria*-like asexual conidiogenous structure. Additionally, both of the fungal sexual morphs have not been determined yet. However, *S.asiatica* can be distinguished from *S.yunnanensis* by its shorter phialides (2.7–8.6 µm) and smaller conidia (1.1–1.8 × 0.8–1.2 µm). Ecologically, *S.asiatica* has been found to parasitize larvae and pupae of Lepidoptera, whereas *S.yunnanensis* is associated with pupae of Limacodidae in cocoons and *Cordyceps* spp. (Wang et al. 2020a). Both morphological and phylogenetic analyses support the idea that this fungus is a distinct species in the genus *Samsoniella*.

#### ﻿ Samsoniella aurantia

Taxon classificationFungiHypocrealesCordycipitaceae

Mongkolsamrit, Noisripoom, Thanakitpipattana, Spatafora & Luangsa-ard

F568AED3-AFC3-5B30-87EB-5281678E1A89

 823786

Fig. 4

##### Type.

Thailand (holotype: BBH 33739; ex-type living culture: TBRC 7271).

**Figure 4.**
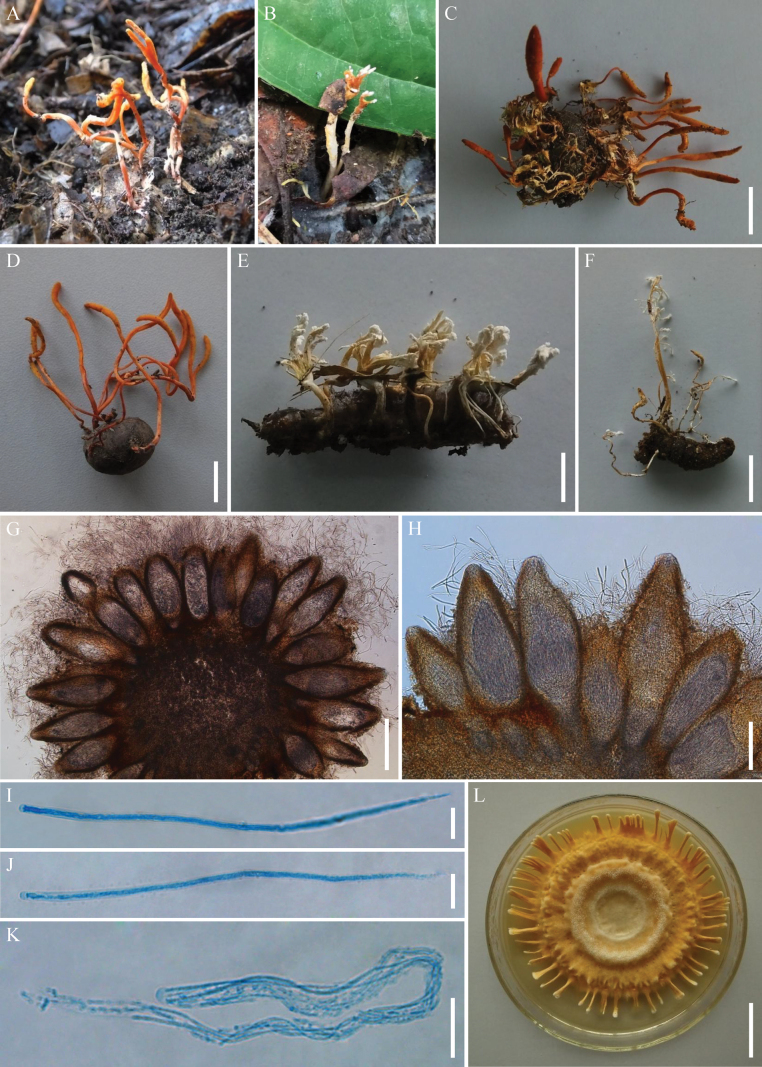
*Samsoniellaaurantia***A, B** perithecial stromata as encountered in the field **C, D** fungus on the pupae of Limacodidae inhabiting cocoons **E, F** synnemata arising from lepidopteran larvae **G, H** Perithecia **I–K** asci **L** colony as obversed on PDA. Scale bars: 10 mm (**C, D, F**); 5 mm (**E**); 200 µm (**G**); 100 µm (**H**); 10 µm (**I, J, K**); 20 mm (**L**).

##### Description.

**Teleomorph**: Stromata arising from lepidopteran insects, gregarious, branched or unbranched, up to 8.8–63.8 mm long. Stipes fleshly, flexuous, yellowish to orange, cylindrical to clavate, 4.1–49.1 × 0.2–2.3 mm. Fertile parts reddish orange, clavate, lateral side usually have a longitudinal section without producing perithecia, 3.8–17.7 × 0.8–4.1 mm. Perithecia crowded, superficial, narrowly ovoid to fusiform, 302.7–449.7 × 105.3–164.9 µm. Asci hyaline, cylindrical, 8‐spored, 92–190 × 1.8–3.6 μm. Apical caps prominent, hemiglobose, 2.1–3.4 µm wide, 1.2–2.3 µm high. Ascospores not observed. **Anamorph**: See Mongkolsamrit et al. (2018). The following descriptions are based on other specimens examined from China. Synnemata arising from lepidopteran larvae. Synnemata erect, flexuous, irregularly branched, white or pale orange, 7.7–32.6 × 0.2–2.1 mm. Stipes cylindrical, producing a mass of conidia at the branches of synnemata, powdery and floccose. Colonies on PDA moderately fast-growing, 26–30 mm diameter in 14 days at 25 °C, light orange to orange, consisting of a basal felt and cottony, sporulating abundantly at the centrum, reverse yellowish, turning deep yellow. Synnemata emerging after 25 days, solitary, unbranched. Size and shape of phialides and conidia similar to that of *S.aurantia* ex-type isolate (TBRC 7271).

##### Distribution.

Chiang Mai Province, Thailand; Guizhou and Yunnan Province, China; Lao Cai Province, Vietnam.

##### Materials examined.

China, Yunnan Province, Zhaotong City, Shuifu County, Taiping Town, Tongluoba National Forest Park (28°24′36″N, 104°9′0″E, 1750 m above sea level), on larvae of Hepialidae living in Qiongzhuea tumidinoda forests, 20 June 2015, collected by Hong Yu (YHH 874, YHH 890–YHH 893; living culture: 874). Vietnam, Lao Cai Province, Sa Pa District, Hoang Lien Mountains (22°21′8″N, 103°46′29″E, 1900 m above sea level), on a pupa of Limacodidae in a cocoon buried in soil, 31 October 2016, Hong Yu (YHH 880, YHH 894; living culture: YFCC 880). Thailand, Chiang Mai Province, Chiang Mai City, Queen Sirikit Botanic Garden (536 m above sea level), on lepidopteran larvae in leaf litter, 26 August 2018, Yao Wang (YHH 895–YHH 896).

##### Commentary.

Numerous species of *Samsoniella* were described originally from asexual morphs, including *S.aurantia* from Thailand (Mongkolsamrit et al. 2018; Wang et al. 2020a; Chen et al. 2022; Wang et al. 2022). Chen et al. (2021) reported *S.aurantia* isolated from a lepidopteran pupa as a new record for China. However, its sexual morph was undetermined in these studies. The present study is the first to report the teleomorph reproductive stage for *S.aurantia*. *Samsoniellaaurantia* has fleshy stromata, clavate fertile parts, superficial perithecia, and cylindrical asci with bola‐shaped ascospores. However, *S.aurantia* differs from other teleomorph species by its phylogenetic placement in the genus by having abundant long stromata extruded from the entire body of lepidopteran insects and by shorter asci measuring 92–190 µm.

#### ﻿ Samsoniella hepiali

Taxon classificationFungiHypocrealesCordycipitaceae

(Q.T. Chen & R.Q. Dai ex R.Q. Dai et al.) H. Yu, R.Q. Dai, Y.B. Wang, Y. Wang & Zhu L. Yang

672867BB-8E9B-5B00-908E-B74FCAFC4EFC

 833114

Fig. 5

##### Type.

China (holotype: IMM 82-2 = CHICMM 82-2; ex-type living culture: ICMM 82-2).

**Figure 5.**
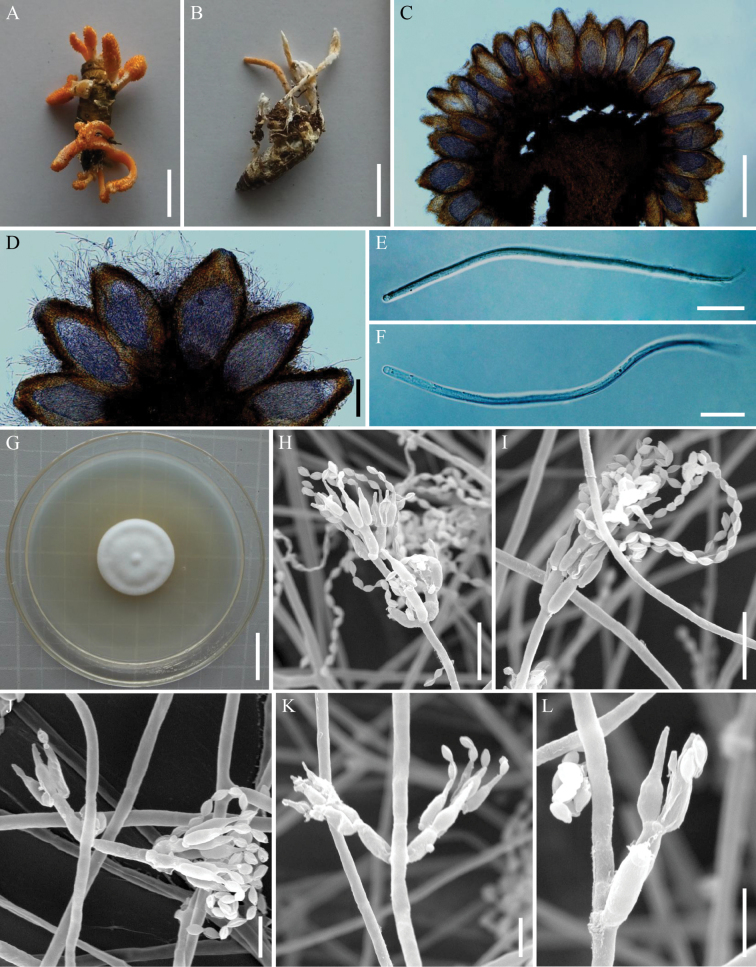
*Samsoniellahepiali***A** stromata of fungus arising from lepidopteran pupa **B** stromata and synnemata arising from lepidopteran pupa **C, D** perithecia **E, F** asci **G** colony as obversed on PDA medium **H–L** conidiophores, phialides and conidia on PDA. Scale bars: 10 mm (**A, B**); 300 µm (**C**); 100 µm (**D**); 20 µm (**E, F**); 20 mm (**G**); 10 µm (**H, I**); 5 µm (**J, K**).

##### Description.

**Teleomorph**: Stromata from the whole body of lepidopteran pupae, gregarious, generally unbranched, up to 5–23 mm long. Stipes fleshly, flexuous or erect, yellowish to orange, cylindrical to clavate, 2.5–15.5 × 0.9–4.6 mm. Fertile parts orange, clavate, lateral side usually have a longitudinal section without producing perithecia, 1.3–8.5 × 0.8–5.2 mm. Perithecia crowded, superficial, narrowly ovoid to fusiform, 277.9–355.3 × 116.3–199.6 µm. Asci hyaline, cylindrical, 8‐spored, 145–300 × 3.5–5 μm. Apical caps prominent, hemiglobose, 2.5–4 µm wide, 2.4–3.2 µm high. Ascospores hyaline, bola‐shaped, septate, 120–240 × 0.8–1.5 μm. **Anamorph**: See Wang et al. (2020a). The following descriptions are based on other specimens examined from Vietnam. Synnemata arising from the whole body of lepidopteran pupae, branched or unbranched, 5–20 mm long. Stipes cylindrical or clavate, 0.6–4.2 mm wide, with powdery conidia at the apex, white to yellowish. Colonies on PDA moderately fast-growing, 32–45 mm diameter in 14 days at 25 °C, white to yellowish, cottony, with high mycelial density, reverse white to pale yellow, turning orange when old. Hyphae smooth-walled, branched, septate, hyaline, 0.9–2.3 µm wide. Conidiophores smooth-walled, cylindrical, solitary, 3.9–10.2 × 1.5–1.9 µm. Phialides on conidiophores verticillate, usually in whorls of two to five, or solitary on hyphae, 5.7–10.9 µm long, basal portion cylindrical to narrowly lageniform, tapering gradually or abruptly toward the apex, from 1.4–1.9 µm wide (base) to 0.5–0.9 µm wide (apex). Conidia smooth and hyaline, fusiform or oval, one-celled, 1.9–2.8 × 1.0–1.6 µm, often in chains. Size and shape of phialides and conidia similar in culture and on natural substratum.

##### Distribution.

Yunnan, Qinghai, Anhui and Guizhou Province, China; Lao Cai Province, Vietnam; Buenos Aires City, Argentina.

##### Materials examined.

Vietnam, Lao Cai Province, Sa Pa District, Hoang Lien Mountains (22°21′10″N, 103°46′29″E, 1989 m above sea level), on pupae of Hepialidae buried in soil, 30 October 2016, collected by Hong Yu (YHH 868, YHH 897–YHH 899; living culture: YFCC 868, YFCC 897–YFCC 899).

##### Commentary.

The strain (YFCC 868) isolated from the pupa of Hepialidae from Vietnam formed a well-supported clade with *S.hepiali* ex-type isolate (ICMM 82-2) (Fig. 2). Based on microscopic observation, the strain YFCC 868 displayed typical morphological characteristics of anamorphs found in species of *Samsoniella*. For YFCC 868, the size and shape of phialides and conidia were similar to those of *S.hepiali* as described by Wang YB et al. (2020b). Both morphological study and phylogenetic analyses supported the isolate YFCC 868 as being *S.hepiali*.

In the current study, the sexual morph of *S.hepiali* was first reported. As for other teleomorph species of *Samsoniella*, *S.hepiali* has fleshy stromata, clavate fertile parts, superficial perithecia, and cylindrical asci with bola‐shaped ascospores. Among these species, only three, namely, *S.cardinalis*, *S.hepiali*, and *S.kunmingensis*, have short stromata (Wang et al. 2020a; Wang et al. 2022). However, *S.hepiali* differs from *S.cardinalis* and *S.kunmingensis* by having abundant stromata extruded from the entire body of lepidopteran insects and by having clavate fertile parts with orange color.

#### ﻿ Samsoniella sapaensis

Taxon classificationFungiHypocrealesCordycipitaceae

H. Yu bis, Y. Wang & Z.Q. Wang sp. nov.

AA2EA5EA-BB45-5040-B080-523B9FDAE1D2

 848023

Fig. 6

##### Etymology.

Named after the location Sa Pa District where the species was collected.

**Figure 6.**
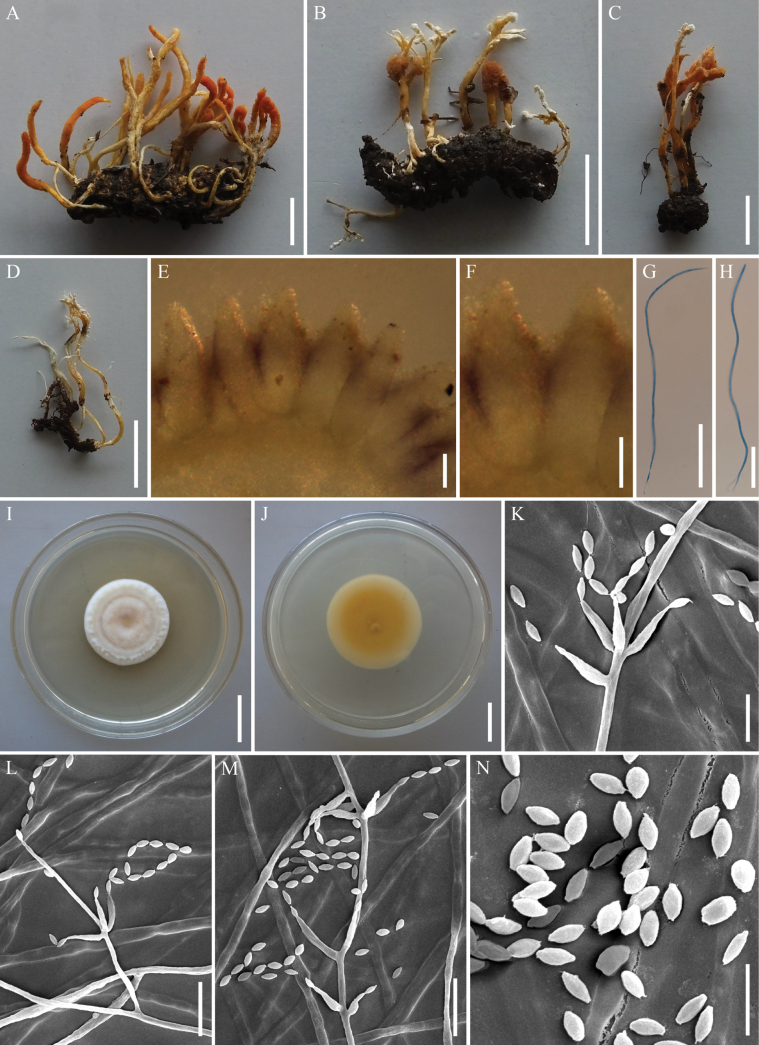
*Samsoniellasapaensis***A** stromata of fungus arising from lepidopteran larva **B, C** stromata and synnemata arising from lepidopteran insects **D** synnemata of fungus **E, F** perithecia **G, H** asci **I, J** colony as obversed and its backside on PDA**K–M** conidiophores, phialides and conidia on PDA**N** conidia on PDA. Scale bars: 10 mm (**A, C**); 5 mm (**B**); 20 mm (**D**); 100 µm (**E, F, G**); 50 µm (**H**); 20 mm (**I**); 30 mm (**J**); 5 µm (**K, N**); 10 µm (**L, M**).

##### Type.

Vietnam, Lao Cai Province, Sa Pa District, Hoang Lien National Park (22°19′30″N, 103°46′50″E, 2178 m above sea level), on a larva of Lepidoptera buried in soil, 26 October 2017, collected by Hong Yu (holotype: YHH 873; ex-type living culture: YFCC 873).

##### Description.

**Teleomorph**: Stromata arising from the whole body of lepidopteran pupae or larvae, gregarious, generally unbranched, up to 22–38 mm long. Stipes fleshly, flexuous, yellowish to orange, cylindrical to clavate, 7.5–14.5 × 0.7–4.6 mm. Fertile parts yellowish to reddish orange, clavate, lateral side usually have a longitudinal section without producing perithecia, 1.5–21.3 × 1.0–2.8 mm. Perithecia crowded, superficial, narrowly ovoid to fusiform, 383.2–412.1 × 125.4–156.9 µm. Asci cylindrical, hyaline, 282.5–444.5 × 2.6–3.9 µm, with a hemispheric apical cap of 1.8–2.2 × 2.6–3.0 µm. Ascospores not observed. **Anamorph**: Synnemata arising from lepidopteran insects. Synnemata flexuous, irregularly branched, white or pale orange, 9–58 × 0.2–1.6 mm, *Isaria*-like morph producing a mass of conidia at the branches of synnemata, powdery and floccose. Colonies on PDA moderately fast-growing, 36–40 mm diameter in 14 days at 25 °C, white to pale pink, cottony, sporulating abundantly, reverse yellow to orange. Hyphae smooth-walled, branched, septate, hyaline, 1.0–1.9 µm wide. Conidiophores smooth-walled, cylindrical, solitary or verticillate, 6.5–17.5 × 1.0–1.6 µm. Phialides verticillate, usually in whorls of two to three, or solitary on hyphae, 2.8–7.6 µm long, basal portion cylindrical to narrowly lageniform, tapering gradually or abruptly toward the apex, from 0.8–1.5 µm wide (base) to 0.6–0.9 µm wide (apex). Conidia smooth and hyaline, fusiform or oval, one-celled, 1.2–1.5 × 0.8–1.0 µm, often in chains. Size and shape of phialides and conidia similar in culture and on natural substratum.

##### Distribution.

At present, known only in Sa Pa District, Lao Cai Province, Vietnam.

##### Additional materials examined.

Vietnam, Lao Cai Province, Sa Pa District (22°21′4″N, 103°46′29″E, 1931 m above sea level), on a pupa of Limacodidae in a cocoon buried in soil, 31 October 2016, collected by Hong Yu (YHH 872; living culture: YFCC 872). Vietnam, Lao Cai Province, Sa Pa District, Hoang Lien National Park (22°19′30″N, 103°46′50″E, 2178 m above sea level), on larvae of Lepidoptera buried in soil, 26 October 2017, collected by Yuan-Bing Wang (YHH 900–YHH 906).

##### Commentary.

*Samsoniellasapaensis* was identified as belonging to *Samsoniella* based on the phylogenetic analyses and was shown to resolve closely to *S.haniana* (Fig. 2). Morphologically, *S.sapaensis* is similar to *S.haniana* in sharing *Isaria*-like asexual conidiogenous structure which produces phialides with cylindrical to narrowly lageniform basal portion, fusiform or oval conidia (Wang et al. 2022). However, two samples of *S.sapaensis* were clustered together and formed a separate clade from *S.haniana* with strong statistical support (BI posterior probabilities = 1, ML bootstrap = 100%). Our morphological observation revealed some differences between them. Phialides on PDA of *S.sapaensis* (2.8–7.6 µm) are shorter than those of *S.haniana* (5.4–12.1 μm). Furthermore, conidia on PDA of *S.sapaensis* (1.2–1.5 × 0.8–1.0 μm) are smaller than those of *S.haniana* (2.3–3.7 × 1.2–2.8 μm).

## ﻿Discussion

DNA sequence data for nr SSU, nr LSU, 3P_*TEF*, *RPB1*, and *RPB2* have been used extensively to explore phylogenetic relationships among *Samsoniella* species in recent years (Mongkolsamrit et al. 2018; Wang et al. 2020a; Wang et al. 2022). However, not all of the recognized species inside this group formed well-supported clades in these five-gene phylogenies. Our results indicate that the ITS sequences (as the primary DNA barcording region for fungi) contain few informative characters for members of the genus (Suppl. material 1). The search for new markers that are appropriate for molecular phylogenetic analysis of *Samsoniella* remains a challenging problem. We attempted to address this in the current study, and to this end, we introduced the 5P_*TEF*, *ACT*, *TUB*, and *MCM7* sequences that had not been previously employed for *Samsoniella* spp.

The ITS, *RPB2*, *ACT*, and *TUB* sequences provided limited valuable information to separate *Samsoniella* spp. In contrast, sequence data for the 3P_*TEF*, 5P_*TEF*, *RPB1*, and *MCM7* loci provided good resolution of *Samsoniella* species (Suppl. material 2). The species delimitations by the tree topologies for the individual loci and the genetic divergence comparisons showed that the 3P_*TEF* sequence data provided the best resolution distinguishing *Samsoniella* spp., followed by 5P*_TEF*, *MCM7*, and *RPB1* sequences (see Suppl. materials 2, 3). Our study introduced valuable sequence data for a single-copy protein-coding gene, *MCM7*. This gene region requires only two primers and is easily amplified. Although the sequence length of the *MCM7* fragment was the shortest among the four loci analyzed in this study, the single-copy protein-coding gene, which was used successfully for determining phylogenetic relationships of *Samsoniella*, provided good resolution for terminal clades in the genus (Suppl. material 2: table S9 and Suppl. material 3: fig. S4). Future studies will benefit from the use of this single locus for the recognition and identification of species in the genus *Samsoniella* and for other fungal species.

In addition to identifying the most of the useful gene regions to accurately identify species of *Samsoniella*, an important goal of this study was to re-establish well-supported boundaries in this genus. Having determined that the 3P_*TEF*, 5P_*TEF*, *MCM7* and *RPB1* regions yielded the best resolution for distinguishing species of *Samsoniella*, a phylogenetic tree based on the combined data (5P_*TEF* + 3P_*TEF* + *RPB1* + *MCM7*) for the genus was generated (Fig. 2). The resulting phylogeny showed well-supported clades for *Samsoniella*, although there was some incongruence with the single-locus phylogenies (Suppl. material 3). According to the phylogenetic tree, 26 out of the 28 molecularly confirmed species in *Samsoniella* were recognized (Fig. 2). Prior studies have delimited *S.formicae* and *S.lepidopterorum* as valid species on the basis of their phylogenies (Chen et al. 2020, 2022); however, our study did not include these sequences because they had abundant missing data, thus their status was not evaluated. These species would require resequencing and further revision to be recognized as supported lineages within the genus *Samsoniella*.

Our multilocus phylogeny demonstrated the cryptic nature of the genus. First, the species status of *S.pseudogunii* is doubtful. From a phylogenetic point of view, *S.pseudogunii* cannot be distinguished from *S.coleopterorum*, being inside the clade of the latter. Regarding the micro-morphology, the two species are also very similar (Chen et al. 2020, 2021). Therefore, we propose that *S.pseudogunii* is a synonym of *S.coleopterorum*. Another example of an ambiguous species is the pair *S.coccinellidicola* / *S.guizhouensis*. These two species were proposed separately from independent studies (*S.coccinellidicola*: Wang et al. 2022, *S.guizhouensis*: Chen et al. 2022); both studies identified their respective new species as a sister taxon to *S.pupicola*. Our phylogenetic trees suggested that *S.guizhouensis* could not be distinguished from *S.coccinellidicola* (Fig. 2). Morphologically, there were no significant differences in the morphological characteristics of anamorphs between the two species except for their host. Because *S.coccinellidicola* was described earlier than *S.guizhouensis* (19 July 2022 vs 12 September 2022), *S.coccinellidicola* should be recommended as the scientific name for this species in accordance with the priority of the international nominating regulations.

Because Mongkolsamrit et al. (2018) first discovered the teleomorph reproductive stage for *S.inthanonensis*, nine members of the genus have been described as teleomorphically typified species (Mongkolsamrit et al. 2018; Wang et al. 2020a; Wang et al. 2022). In this study, the sexual morph of *S.aurantia*, *S.hepiali*, and *S.sapaensis* sp. nov. were first reported. However, the observation of the teleomorph in nature is relatively rare for the majority of *Samsoniella* species existing as anamorphs. Due to their rarity, each teleomorph specimen is precious (Wang et al. 2020b). The teleomorph specimens of some species, such as *S.hepiali*, are of great economic and medical value in integrated phylogenetic, developmental, and mating studies. These studies are not only helpful in providing insight into systematic and life history studies, but they are also meaningful to prevent cultivated strains from degeneration (Rehner and Buckley 2005; Wang et al. 2020b).

## Supplementary Material

XML Treatment for Samsoniella asiatica

XML Treatment for Samsoniella aurantia

XML Treatment for Samsoniella hepiali

XML Treatment for Samsoniella sapaensis

## ﻿Additional information

### Author ORCIDs

Yao Wang https://orcid.org/0000-0002-1262-6700

Hong Yu https://orcid.org/0000-0002-2149-5714

### Data availability

All of the data that support the findings of this study are available in the main text or Supplementary Information.

## Supplementary materials

Supplementary material 1 Specimen information and GenBank accession numbers Data type docx Explanation note **table S1.** Specimen information and GenBank accession numbers for sequences used in the analyses of a five-locus (nr SSU, nr LSU, 3P_*TEF*, *RPB1*, and *RPB2*) dataset. https://binary.pensoft.net/file/915717This dataset is made available under the Open Database License (http://opendatacommons.org/licenses/odbl/1.0/). The Open Database License (ODbL) is a license agreement intended to allow users to freely share, modify, and use this Dataset while maintaining this same freedom for others, provided that the original source and author(s) are credited.Yao Wang, Zhi-Qin Wang, Chinnapan Thanarut, Van-Minh Dao, Yuan-Bing Wang, Hong Yu

Supplementary material 2 Pairwise genetic distance matrix of *Samsoniella* species Data type docx Explanation note **table S2.** Pairwise genetic distance matrix of *Samsoniella* species for ITS sequences. **table S3.** Pairwise genetic distance matrix of *Samsoniella* species for 3P-*TEF* sequences. **table S4.** Pairwise genetic distance matrix of *Samsoniella* species for 5P-*TEF* sequences. **table S5.** Pairwise genetic distance matrix of *Samsoniella* species for *RPB1* sequences. **table S6.** Pairwise genetic distance matrix of *Samsoniella* species for *RPB2* sequences. **table S7.** Pairwise genetic distance matrix of *Samsoniella* species for *ACT* sequences. **table S8.** Pairwise genetic distance matrix of *Samsoniella* species for *TUB* sequences. **table S9.** Pairwise genetic distance matrix of *Samsoniella* species for *MCM7* sequences. https://binary.pensoft.net/file/915718This dataset is made available under the Open Database License (http://opendatacommons.org/licenses/odbl/1.0/). The Open Database License (ODbL) is a license agreement intended to allow users to freely share, modify, and use this Dataset while maintaining this same freedom for others, provided that the original source and author(s) are credited.Yao Wang, Zhi-Qin Wang, Chinnapan Thanarut, Van-Minh Dao, Yuan-Bing Wang, Hong Yu

Supplementary material 3 Phylogenetic tree of *Samsoniella* Data type docx Explanation note **figure S1.** Phylogenetic tree of *Samsoniella* based on Maximum Likelihood (ML) analysis from the 5P-*TEF* sequences. Statistical support values (≥50%) are shown at the nodes for ML boostrap support. **figure S2.** Phylogenetic tree of *Samsoniella* based on Maximum Likelihood (ML) analysis from the 3P-*TEF* sequences. Statistical support values (≥50%) are shown at the nodes for ML boostrap support. **figure S3.** Phylogenetic tree of *Samsoniella* based on Maximum Likelihood (ML) analysis from the *RPB1* sequences. Statistical support values (≥50%) are shown at the nodes for ML boostrap support. **figure S4.** Phylogenetic tree of *Samsoniella* based on Maximum Likelihood (ML) analysis from the *MCM7* sequences. Statistical support values (≥50%) are shown at the nodes for ML boostrap support. https://binary.pensoft.net/file/915719This dataset is made available under the Open Database License (http://opendatacommons.org/licenses/odbl/1.0/). The Open Database License (ODbL) is a license agreement intended to allow users to freely share, modify, and use this Dataset while maintaining this same freedom for others, provided that the original source and author(s) are credited.Yao Wang, Zhi-Qin Wang, Chinnapan Thanarut, Van-Minh Dao, Yuan-Bing Wang, Hong Yu

Supplementary material 4 Highlights Data type docx https://binary.pensoft.net/file/915720This dataset is made available under the Open Database License (http://opendatacommons.org/licenses/odbl/1.0/). The Open Database License (ODbL) is a license agreement intended to allow users to freely share, modify, and use this Dataset while maintaining this same freedom for others, provided that the original source and author(s) are credited.Yao Wang, Zhi-Qin Wang, Chinnapan Thanarut, Van-Minh Dao, Yuan-Bing Wang, Hong Yu
