# Supplementary material for: ﻿Phylogeny and species delimitations in the economically, medically, and ecologically important genus Samsoniella (Cordycipitaceae, Hypocreales)
Source: MycoKeys. 2023 Oct 3;99:227–50. doi: 10.3897/mycokeys.99.106474 (PMC10565569; doi:10.3897/mycokeys.99.106474)
Supplement: Supplementary material 1 — Specimen information and GenBank accession numbers [file mycokeys-99-227-s001.docx]

**Table S1** Specimen information and GenBank accession numbers for sequences used in the analyses of a five-locus (nr*SSU*, nr*LSU*, 3P_*TEF*, *RPB1*, and *RPB2*) dataset.

| **Taxon** | **Voucher information** | **Host/Substrate** | **GenBank accession number** | | | | |
| --- | --- | --- | --- | --- | --- | --- | --- |
|  |  |  | **nr*SSU*** | **nr*LSU*** | **3P*_TEF*** | ***RPB1*** | ***RPB2*** |
| *Akanthomyces attenuatus* | CBS 402.78 | Leaf litter | AF339614 | AF339565 | EF468782 | EF468888 | EF468935 |
| *Akanthomyces coccidioperitheciatus* | NHJ 6709 | Araneae | EU369110 | EU369042 | EU369025 | EU369067 | EU369086 |
| *Akanthomyces dipterigenus* | CBS 126.27 | Hemiptera: Monophlebidae | AF339605 | AF339556 | KM283820 | KR064300 | KR064303 |
| *Akanthomyces lecanii* | CBS 101247 | Hemiptera: Coccidae | AF339604 | AF339555 | DQ522359 | DQ522407 | DQ522466 |
| *Akanthomyces muscarius* | CBS 143.62 | Hemiptera: Aleyrodidae | KM283774 | KM283798 | KM283821 | KM283841 | KM283863 |
| *Akanthomyces sabanensis* | ANDES-F 1024 | Hemiptera: Coccidae | KC633251 | KC875225 | KC633266 |  | KC633249 |
| *Akanthomyces sulphureus* | TBRC 7248 | Araneae |  | MF140722 | MF140843 | MF140787 | MF140812 |
| *Akanthomyces tuberculatus* | BCC 16819 | Lepidopteran adult | MF416600 | MF416546 | MF416490 | MF416647 | MF416444 |
| *Akanthomyces waltergamsii* | TBRC 7251 | Araneae |  | MF140713 | MF140833 | MF140781 | MF140805 |
| *Akanthomyces waltergamsii* | TBRC 7252 | Araneae |  | MF140714 | MF140834 | MF140782 | MF140806 |
| *Amphichorda felina* | YFCC 850 | Bird droppings | MW181774 | MW173986 | MW168227 | MW168193 | MW168210 |
| *Amphichorda felina* | YFCC 851 | Bird droppings | MW181775 | MW173987 | MW168228 | MW168194 | MW168211 |
| *Amphichorda guana* | CGMCC 3.17908 | Bat guano | KY883262 | KU746711 | KX855211 | KY883202 | KY883228 |
| *Amphichorda guana* | CGMCC 3.17909 | Bat guano | KY883263 | KU746712 | KX855212 | KY883203 |  |
| *Ascopolyporus polychrous* | P.C. 546 | Plant |  | DQ118737 | DQ118745 | DQ127236 |  |
| *Ascopolyporus villosus* | ARSEF 6355 | Plant |  | AY886544 | DQ118750 | DQ127241 |  |
| *Beauveria acridophila* | HUA 179219 | Orthoptera: Acrididae |  | JQ895541 | JQ958613 | JX003857 | JX003841 |
| *Beauveria acridophila* | HUA 179220 | Orthoptera: Acrididae | JQ895527 | JQ895536 | JQ958614 | JX003852 | JX003842 |
| *Beauveria amorpha* | ARSEF 2641 | Hymenoptera: Formicidae |  |  | AY531917 | HQ880880 | HQ880952 |
| *Beauveria araneola* | GZAC 150317 | Araneae |  |  | KT961699 | KT961701 |  |
| *Beauveria asiatica* | ARSEF 4850 | Coleoptera: Cerambycidae |  |  | AY531937 | HQ880859 | HQ880931 |
| *Beauveria asiatica* | YFCC 5600 | Coleoptera: Cerambycidae | MN576770 | MN576826 | MN576996 | MN576886 | MN576940 |
| *Beauveria australis* | ARSEF 4598 | Soil |  |  | HQ880995 | HQ880861 | HQ880933 |
| *Beauveria baoshanensis* | CCTCC AF 2018011 | Coleoptera: Chrysomelidae | MG642882 | MG642840 | MG642897 | MG642854 | MG642867 |
| *Beauveria bassiana* | ARSEF 1564 | Lepidoptera: Arctiidae |  |  | HQ880974 | HQ880833 | HQ880905 |
| *Beauveria bassiana* | YFCC 3369 | Coleoptera: Scarabaeidae | MN576768 | MN576824 | MN576994 | MN576884 | MN576938 |
| *Beauveria blattidicola* | MCA 1727 | Blattodea: Blattidae | MF416593 | MF416539 | MF416483 | MF416640 |  |
| *Beauveria blattidicola* | MCA 1814 | Blattodea: Blattidae | MF416594 | MF416540 | MF416484 | MF416641 |  |
| *Beauveria brongniartii* | ARSEF 617 | Coleoptera: Scarabaeidae | AB027335 | AB027381 | HQ880991 | HQ880854 | HQ880926 |
| *Beauveria brongniartii* | YFCC 3240 | Coleoptera: Scarabaeidae | MN576769 | MN576825 | MN576995 | MN576885 | MN576939 |
| *Beauveria caledonica* | ARSEF 2567 | Soil | AF339570 | AF339520 | EF469057 | HQ880889 | HQ880961 |
| *Beauveria caledonica* | YFCC 7025 | Coleoptera: Cerambycidae | MN576771 | MN576827 | MN576997 | MN576887 | MN576941 |
| *Beauveria diapheromeriphila* | QCNE 186272 | Phasmatodea: Diapheromeridae | JQ895530 | JQ895534 | JQ958610 | JX003848 |  |
| *Beauveria diapheromeriphila* | QCNE 186714 | Phasmatodea: Diapheromeridae | MF416601 | MF416547 | MF416491 | MF416648 |  |
| *Beauveria hoplocheli* | Bt116 | Coleoptera: Melolonthidae |  |  | KC339703 | KM453957 | KM453966 |
| *Beauveria hoplocheli* | MNHN-RF-06107 | Coleoptera: Melolonthidae |  |  | KC339702 | KM453954 | KM453963 |
| *Beauveria kipukae* | ARSEF 7032 | Homoptera: Delphacidae |  |  | HQ881005 | HQ880875 | HQ880947 |
| *Beauveria lii* | ARSEF 11741 | Coleoptera: Coccinellidae |  |  | JN689371 | JN689374 | JN689370 |
| *Beauveria locustiphila* | TS881 | Orthoptera: Romaleidae | JQ895525 | JQ895535 | JQ958619 | JX003847 | JX003845 |
| *Beauveria majiangensis* | GZAC GZU12141 | Coleoptera: Scarabaeoidea |  |  | MG052640 | MG052644 |  |
| *Beauveria majiangensis* | YFCC 852 | Hemiptera: Pentatomidae | MW181776 | MW173988 | MW168229 | MW168195 | MW168212 |
| *Beauveria malawiensis* | ARSEF 7760 | Coleoptera: Cerambycidae |  |  | DQ376246 | HQ880897 | HQ880969 |
| *Beauveria malawiensis* | YFCC 853 | Coleoptera: Scarabaeoidea | MW181777 | MW173989 | MW168230 | MW168196 | MW168213 |
| *Beauveria medogensis* | 2898 | Soil |  |  | KU994833 | KU994835 | KU994834 |
| *Beauveria medogensis* | YFCC 854 | Coleopteran adult | MW181778 | MW173990 | MW168231 | MW168197 | MW168214 |
| *Beauveria peruviensis* | ARSEF 14196 | Coleoptera: Curculionidae |  |  | MN094781 | MN100118 |  |
| *Beauveria peruviensis* | UTRF35 | Coleoptera: Curculionidae |  |  | MN094771 | MN100115 |  |
| *Beauveria polyrhachicola* | YFCC 859 | Hymenoptera: Formicidae | MW181783 | MW173995 | MW168236 | MW168202 | MW168219 |
| *Beauveria polyrhachicola* | YHH 859 | Hymenoptera: Formicidae | MW181784 | MW173996 | MW168237 | MW168203 | MW168220 |
| *Beauveria pseudobassiana* | ARSEF 3405 | Lepidoptera: Tortricidae |  |  | AY531931 | HQ880864 | HQ880936 |
| *Beauveria pseudobassiana* | YFCC 1806007 | Coleoptera: Scarabaeidae | MN523495 | MN523524 | MN523553 | MN523582 | MN523611 |
| *Beauveria scarabaeidicola* | ARSEF 5689 | Coleoptera: Scarabaeidae | AF339574 | AF339524 | DQ522335 | DQ522380 | DQ522431 |
| *Beauveria sinensis* | BUB 504 | Orthoptera: Grylloidea | MG642880 | MG642838 | MG642895 | MG642852 | MG642865 |
| *Beauveria sinensis* | RCEF 3903 | Lepidoptera: Geometridae |  |  | HQ270151 | JX524283 | JX524284 |
| *Beauveria songmingensis* | YFCC 860 | Coleoptera: Scarabaeidae | MW181785 | MW173997 | MW168238 | MW168204 | MW168221 |
| *Beauveria songmingensis* | YFCC 861 | Coleoptera: Scarabaeidae | MW181786 | MW173998 | MW168239 | MW168205 | MW168222 |
| *Beauveria staphylinidicola* | ARSEF 5718 | Coleoptera: Staphylinidae | EF468981 | EF468836 | EF468776 | EF468881 |  |
| *Beauveria staphylinidicola* | YFCC 855 | Coleoptera: Cerambycidae | MW181779 | MW173991 | MW168232 | MW168198 | MW168215 |
| *Beauveria subscarabaeidicola* | YFCC 863 | Coleoptera: Scarabaeidae | MW181788 | MW174000 | MW168241 | MW168207 | MW168224 |
| *Beauveria subscarabaeidicola* | YFCC 864 | Coleoptera: Scarabaeidae | MW181789 | MW174001 | MW168242 | MW168208 | MW168225 |
| *Beauveria varroae* | ARSEF 8257 | Coleoptera: Curculionidae |  |  | HQ881002 | HQ880872 | HQ880944 |
| *Beauveria vermiconia* | ARSEF 2922 | Soil |  |  | AY531920 | HQ880894 | HQ880966 |
| *Beauveria yunnanensis* | CCTCC AF 2018010 | Lepidopteran pupa | MG642885 | MG642843 | MG642900 | MG642857 | MG642870 |
| *Beauveria yunnanensis* | YFCC 3105 | Coleoptera: Scarabaeidae | MN576773 | MN576829 | MN576999 | MN576889 | MN576943 |
| *Blackwellomyces cardinalis* | OSC 93609 | Lepidoptera: Tineidae | AY184973 | AY184962 | DQ522325 | DQ522370 | DQ522422 |
| *Blackwellomyces cardinalis* | OSC 93610 | Lepidoptera: Tineidae | AY184974 | AY184963 | EF469059 | EF469088 | EF469106 |
| *Blackwellomyces pseudomilitaris* | BCC 1919 | Lepidopteran larva | MF416588 | MF416534 | MF416478 |  | MF416440 |
| *Blackwellomyces pseudomilitaris* | BCC 2091 | Lepidopteran larva | MF416589 | MF416535 | MF416479 |  | MF416441 |
| *Cordyceps amoene-rosea* | CBS 107.73 | Coleopteran pupa | AY526464 | MF416550 | MF416494 | MF416651 | MF416445 |
| *Cordyceps bifusispora* | EFCC 5690 | Lepidopteran pupa | EF468952 | EF468806 | EF468746 | EF468854 | EF468909 |
| *Cordyceps bifusispora* | EFCC 8260 | Lepidopteran pupa | EF468953 | EF468807 | EF468747 | EF468855 | EF468910 |
| *Cordyceps blackwelliae* | TBRC 7256 | Coleopteran larva |  | MF140702 | MF140822 | MF140771 | MF140795 |
| *Cordyceps blackwelliae* | YFCC 856 | Lepidopteran larva | MW181780 | MW173992 | MW168233 | MW168199 | MW168216 |
| *Cordyceps caloceroides* | MCA 2249 | Araneae | MF416578 | MF416525 | MF416470 | MF416632 |  |
| *Cordyceps cateniobliqua* | CBS 153.83 | Lepidoptera: Tortricidae | AY526466 |  | JQ425688 |  | MG665236 |
| *Cordyceps cateniobliqua* | YFCC 3367 | Coleopteran adult | MN576765 | MN576821 | MN576991 | MN576881 | MN576935 |
| *Cordyceps chanhua* | RCEF HP090724-31 | Hemiptera: Cicadidae | MF416605 | MF416552 | MF416496 | MF416653 | MF416447 |
| *Cordyceps chiangdaoensis* | TBRC 7274 | Coleoptera |  | MF140732 | KT261403 |  |  |
| *Cordyceps chiangdaoensis* | YFCC 857 | Coleoptera: Elateridae | MW181781 | MW173993 | MW168234 | MW168200 | MW168217 |
| *Cordyceps coleopterorum* | CBS 110.73 | Coleopteran larva | JF415965 | JF415988 | JF416028 | JN049903 | JF416006 |
| *Cordyceps exasperata* | MCA 2288 | Lepidopteran larva | MF416592 | MF416538 | MF416482 | MF416639 |  |
| *Cordyceps farinosa* | CBS 111113 | Unknown | AY526474 | MF416554 | MF416499 | MF416656 | MF416450 |
| *Cordyceps fumosorosea* | CBS 244.31 | Butter | MF416609 | MF416557 | MF416503 | MF416660 | MF416454 |
| *Cordyceps fumosorosea* | YFCC 4561 | Lepidoptera | MN576761 | MN576817 | MN576987 | MN576877 | MN576931 |
| *Cordyceps javanica* | CBS 134.22 | Coleoptera | MF416610 | MF416558 | MF416504 | MF416661 | MF416455 |
| *Cordyceps javanica* | TBRC 7259 | Lepidoptera |  | MF140711 | MF140831 | MF140780 | MF140804 |
| *Cordyceps javanica* | YFCC 3368 | Lepidoptera | MN576767 | MN576823 | MN576993 | MN576883 | MN576937 |
| *Cordyceps kyusyuensis* | EFCC 5886 | Lepidopteran pupa | EF468960 | EF468813 | EF468754 | EF468863 | EF468917 |
| *Cordyceps militaris* | YFCC 6587 | Lepidopteran pupa | MN576762 | MN576818 | MN576988 | MN576878 | MN576932 |
| *Cordyceps ninchukispora* | EGS 38.165 | Plant(*Beilschmiedia erythrophloia*) | EF468991 | EF468846 | EF468795 | EF468900 |  |
| *Cordyceps ninchukispora* | EGS 38.166 | Plant(*Beilschmiedia erythrophloia*) | EF468992 | EF468847 | EF468794 | EF468901 |  |
| *Cordyceps oncoperae* | ARSEF 4358 | Lepidoptera: Hepialidae | AF339581 | AF339532 | EF468785 | EF468891 | EF468936 |
| *Cordyceps polyarthra* | MCA 996 | Lepidoptera | MF416597 | MF416543 | MF416487 | MF416644 |  |
| *Cordyceps polyarthra* | MCA 1009 | Lepidoptera | MF416598 | MF416544 | MF416488 | MF416645 |  |
| *Cordyceps pruinosa* | ARSEF 5413 | Lepidoptera: Limacodidae | AY184979 | AY184968 | DQ522351 | DQ522397 | DQ522451 |
| *Cordyceps rosea* | spat 09-053 | Lepidopteran larva | MF416590 | MF416536 | MF416480 | MF416637 | MF416442 |
| *Cordyceps tenuipes* | ARSEF 5135 | Lepidopteran pupa | MF416612 | JF415980 | JF416020 | JN049896 | JF416000 |
| *Gibellula leiopus* | BCC 16025 | Araneae | MF416602 | MF416548 | MF416492 | MF416649 |  |
| *Gibellula longispora* | NHJ 12014 | Araneae | EU369098 |  | EU369017 | EU369055 | EU369075 |
| *Gibellula pulchra* | NHJ 10808 | Araneae | EU369099 | EU369035 | EU369018 | EU369056 | EU369076 |
| *Hevansia arachnophilus* | NHJ 10469 | Araneae | EU369090 | EU369031 | EU369008 | EU369047 |  |
| *Hevansia cinereus* | NHJ 3510 | Araneae | EU369091 |  | EU369009 | EU369048 | EU369070 |
| *Hevansia nelumboides* | BCC 41864 | Araneae | JN201863 | JN201873 | JN201867 |  |  |
| *Hevansia novoguineensis* | NHJ 11923 | Araneae | EU369095 | EU369032 | EU369013 | EU369052 | EU369072 |
| *Samsoniella alboaurantium* | CBS 240.32 | Lepidopteran pupa | JF415958 | JF415979 | JF416019 | JN049895 | JF415999 |
| *Samsoniella alboaurantium* | CBS 262.58 | Soil | AB023943 | AB080087 | MF416497 | MF416654 | MF416448 |
| *Samsoniella alpina* | YFCC 5818 | Lepidopteran larva (*Hepialus baimaensis*) | MN576753 | MN576809 | MN576979 | MN576869 | MN576923 |
| *Samsoniella alpina* | YFCC 5831 | Lepidopteran larva (*Hepialus baimaensis*) | MN576754 | MN576810 | MN576980 | MN576870 | MN576924 |
| *Samsoniella antleroides* | YFCC 6016 | Lepidopteran larva (Noctuidae sp.) | MN576747 | MN576803 | MN576973 | MN576863 | MN576917 |
| *Samsoniella antleroides* | YFCC 6113 | Lepidopteran larva (Noctuidae sp.) | MN576748 | MN576804 | MN576974 | MN576864 | MN576918 |
| ***Samsoniella asiatica*** | **YFCC 869** | **Lepidopteran pupa** | **OQ476497** | **OQ476505** | **OQ506153** | **OQ506195** | **OQ506187** |
| ***Samsoniella asiatica*** | **YFCC 870** | **Lepidopteran larva (*Spilosoma* sp.)** | **OQ476498** | **OQ476506** | **OQ506154** | **OQ506196** | **OQ506188** |
| ***Samsoniella asiatica*** | **YFCC 871** | **Lepidopteran larva (Noctuidae sp.)** | **OQ476499** | **OQ476507** | **OQ506155** | **OQ506197** | **OQ506189** |
| *Samsoniella aurantia* | TBRC 7271 | Lepidoptera |  | MF140728 | MF140846 | MF140791 | MF140818 |
| ***Samsoniella aurantia*** | **YFCC 874** | **Lepidopteran larva (Hepialidae sp.)** | **OQ476501** | **OQ476509** | **OQ506157** | **OQ506199** | **OQ506191** |
| ***Samsoniella aurantia*** | **YFCC 880** | **Lepidopteran larva (Limacodidae sp.)** | **OQ476500** | **OQ476508** | **OQ506156** | **OQ506198** | **OQ506190** |
| *Samsoniella cardinalis* | YFCC 5830 | Lepidopteran pupa (Limacodidae sp.) | MN576732 | MN576788 | MN576958 | MN576848 | MN576902 |
| *Samsoniella cardinalis* | YFCC 6144 | Lepidopteran pupa (Limacodidae sp.) | MN576730 | MN576786 | MN576956 | MN576846 | MN576900 |
| *Samsoniella coleopterorum* | A19501 | Coleoptera: Curculionidae |  |  | MN101586 | MT642600 | MN101585 |
| *Samsoniella cristata* | YFCC 6023 | Lepidopteran pupa (Saturniidae sp.) | MN576736 | MN576792 | MN576962 | MN576852 | MN576906 |
| *Samsoniella cristata* | YFCC 7004 | Lepidopteran pupa (Saturniidae sp.) | MN576737 | MN576793 | MN576963 | MN576853 | MN576907 |
| *Samsoniella hepiali* | ICMM 82-2 | Fungi (*Ophiocordyceps sinensis*) | MN576738 | MN576794 | MN576964 | MN576854 | MN576908 |
| ***Samsoniella hepiali*** | **YFCC 868** | **Lepidopteran pupa (Hepialidae sp.)** | **OQ476502** | **OQ476510** | **OQ506158** | **OQ506200** | **OQ506192** |
| *Samsoniella hepiali* | YFCC 2702 | Fungi (*Ophiocordyceps sinensis*) | MN576740 | MN576796 | MN576966 | MN576856 | MN576910 |
| *Samsoniella hymenopterorum* | A19521 | Hymenoptera: Vespidae |  |  | MN101588 | MT642603 | MT642604 |
| *Samsoniella hymenopterorum* | A19522 | Hymenoptera: Vespidae |  |  | MN101591 | MN101589 | MN101590 |
| *Samsoniella inthanonensis* | TBRC 7915 | Lepidopteran pupa |  | MF140725 | MF140849 | MF140790 | MF140815 |
| *Samsoniella inthanonensis* | TBRC 7916 | Lepidopteran pupa |  | MF140724 | MF140848 | MF140789 | MF140814 |
| *Samsoniella kunmingensis* | YHH 16002 | Lepidopteran pupa | MN576746 | MN576802 | MN576972 | MN576862 | MN576916 |
| *Samsoniella lanmaoa* | YFCC 6148 | Lepidopteran pupa | MN576733 | MN576789 | MN576959 | MN576849 | MN576903 |
| *Samsoniella lanmaoa* | YFCC 6193 | Lepidopteran pupa | MN576734 | MN576790 | MN576960 | MN576850 | MN576904 |
| *Samsoniella pseudogunii* | GY407201 | Lepidopteran larva |  | MZ827010 | MZ855233 |  | MZ855239 |
| *Samsoniella pseudogunii* | GY407202 | Lepidopteran larva |  | MZ831865 | MZ855234 |  | MZ855240 |
| *Samsoniella pupicola* | DY101681 | Lepidopteran pupa |  | MZ827009 | MZ855231 |  | MZ855237 |
| *Samsoniella pupicola* | DY101682 | Lepidopteran pupa |  | MZ827635 | MZ855232 |  | MZ855238 |
| *Samsoniella ramosa* | YFCC 6020 | Lepidopteran pupa (Limacodidae sp.) | MN576749 | MN576805 | MN576975 | MN576865 | MN576919 |
| ***Samsoniella sapaensis*** | **YFCC 872** | **Lepidopteran pupa (Limacodidae sp.)** | **OQ476495** | **OQ476503** | **OQ506151** | **OQ506193** | **OQ506185** |
| ***Samsoniella sapaensis*** | **YFCC 873** | **Lepidopteran larva** | **OQ476496** | **OQ476504** | **OQ506152** | **OQ506194** | **OQ506186** |
| *Samsoniella tortricidae* | YFCC 6013 | Lepidopteran pupa (Tortricidae sp.) | MN576751 | MN576807 | MN576977 | MN576867 | MN576921 |
| *Samsoniella tortricidae* | YFCC 6131 | Lepidopteran pupa (Tortricidae sp.) | MN576750 | MN576806 | MN576976 | MN576866 | MN576920 |
| *Samsoniella yunnanensis* | YFCC 1527 | Fungi (*Cordyceps cicadae*) | MN576756 | MN576812 | MN576982 | MN576872 | MN576926 |
| *Samsoniella yunnanensis* | YFCC 1824 | Fungi (*Cordyceps cicadae*) | MN576757 | MN576813 | MN576983 | MN576873 | MN576927 |
| *Simplicillium formicae* | MFLUCC 18-1379 | Hymenoptera: Formicidae | MK765046 | MK766512 | MK926451 | MK882623 |  |
| *Simplicillium lamellicola* | CBS 116.25 | Fungi (*Agaricus bisporus*) | AF339601 | AF339552 | DQ522356 | DQ522404 | DQ522462 |
| *Simplicillium lanosoniveum* | CBS 704.86 | Fungi (*Hemileia vastatrix*) | AF339602 | AF339553 | DQ522358 | DQ522406 | DQ522464 |
| *Simplicillium obclavatum* | CBS 311.74 | Air above sugarcane field | AF339567 | AF339517 | EF468798 |  |  |
| *Torrubiella ratticaudata* | ARSEF 1915 | Araneae | DQ522562 | DQ518777 | DQ522360 | DQ522408 | DQ522467 |
| *Trichoderma deliquescens* | ATCC 208838 | On decorticated conifer wood | AF543768 | AF543791 | AF543781 | AY489662 | DQ522446 |
| *Trichoderma stercorarium* | ATCC 62321 | Cow dung | AF543769 | AF543792 | AF543782 | AY489633 | EF469103 |

Boldface: data generated in this study.
