# Supplementary material for: ﻿Phylogeny and species delimitations in the economically, medically, and ecologically important genus Samsoniella (Cordycipitaceae, Hypocreales)
Source: MycoKeys. 2023 Oct 3;99:227–50. doi: 10.3897/mycokeys.99.106474 (PMC10565569; doi:10.3897/mycokeys.99.106474)
Supplement: Supplementary material 2 — Pairwise genetic distance matrix of Samsoniella species [file mycokeys-99-227-s002.docx]

**Table S2** Pairwise genetic distance matrix of *Samsoniella* species for ITS sequences.

| Taxon | 1 | 2 | 3 | 4 | 5 | 6 | 7 | 8 | 9 | 10 | 11 |
| --- | --- | --- | --- | --- | --- | --- | --- | --- | --- | --- | --- |
| 1 (*Samsoniella alpina* YFCC 5818) |  |  |  |  |  |  |  |  |  |  |  |
| 2 (*Samsoniella antleroides* YFCC 6113) | 0.002 |  |  |  |  |  |  |  |  |  |  |
| 3 (*Samsoniella asiatica* YFCC 870 ) | 0.003 | 0.005 |  |  |  |  |  |  |  |  |  |
| 4 (*Samsoniella aurantia* YFCC 874) | 0.005 | 0.007 | 0.005 |  |  |  |  |  |  |  |  |
| 5 (*Samsoniella cardinalis* YFCC 6144) | 0.000 | 0.002 | 0.003 | 0.005 |  |  |  |  |  |  |  |
| 6 (*Samsoniella cristata* YFCC 7004) | 0.002 | 0.003 | 0.005 | 0.007 | 0.002 |  |  |  |  |  |  |
| 7 (*Samsoniella hepiali* ICMM 82-2) | 0.000 | 0.002 | 0.003 | 0.005 | 0.000 | 0.002 |  |  |  |  |  |
| 8 (*Samsoniella lanmaoa* YFCC 6148) | 0.002 | 0.003 | 0.005 | 0.007 | 0.002 | 0.003 | 0.002 |  |  |  |  |
| 9 (*Samsoniella sapaensis* YFCC 873) | 0.005 | 0.007 | 0.005 | 0.000 | 0.005 | 0.007 | 0.005 | 0.007 |  |  |  |
| 10 (*Samsoniella tortricidae* YFCC 6131) | 0.002 | 0.003 | 0.005 | 0.007 | 0.002 | 0.003 | 0.002 | 0.000 | 0.007 |  |  |
| 11 (*Samsoniella yunnanensis* YFCC 1527) | 0.002 | 0.003 | 0.002 | 0.003 | 0.002 | 0.003 | 0.002 | 0.003 | 0.003 | 0.003 |  |

The mean threshold (p-distances) is 0.003.

**Table S3** Pairwise genetic distance matrix of *Samsoniella* species for 3P-*TEF* sequences.

| Taxon | 1 | 2 | 3 | 4 | 5 | 6 | 7 | 8 | 9 | 10 | 11 |
| --- | --- | --- | --- | --- | --- | --- | --- | --- | --- | --- | --- |
| 1 (*Samsoniella alpina* YFCC 5818) |  |  |  |  |  |  |  |  |  |  |  |
| 2 (*Samsoniella antleroides* YFCC 6113) | 0.028 |  |  |  |  |  |  |  |  |  |  |
| 3 (*Samsoniella asiatica* YFCC 870 ) | 0.029 | 0.034 |  |  |  |  |  |  |  |  |  |
| 4 (*Samsoniella aurantia* YFCC 874) | 0.028 | 0.007 | 0.031 |  |  |  |  |  |  |  |  |
| 5 (*Samsoniella cardinalis* YFCC 6144) | 0.005 | 0.030 | 0.029 | 0.029 |  |  |  |  |  |  |  |
| 6 (*Samsoniella cristata* YFCC 7004) | 0.030 | 0.003 | 0.033 | 0.005 | 0.030 |  |  |  |  |  |  |
| 7 (*Samsoniella hepiali* ICMM 82-2) | 0.027 | 0.039 | 0.024 | 0.039 | 0.029 | 0.040 |  |  |  |  |  |
| 8 (*Samsoniella lanmaoa* YFCC 6148) | 0.035 | 0.010 | 0.035 | 0.008 | 0.036 | 0.009 | 0.045 |  |  |  |  |
| 9 (*Samsoniella sapaensis* YFCC 873) | 0.022 | 0.019 | 0.031 | 0.017 | 0.028 | 0.021 | 0.036 | 0.024 |  |  |  |
| 10 (*Samsoniella tortricidae* YFCC 6131) | 0.030 | 0.003 | 0.035 | 0.010 | 0.031 | 0.004 | 0.040 | 0.013 | 0.020 |  |  |
| 11 (*Samsoniella yunnanensis* YFCC 1527) | 0.027 | 0.035 | 0.017 | 0.035 | 0.029 | 0.036 | 0.017 | 0.039 | 0.033 | 0.034 |  |

The mean threshold (p-distances) is 0.026.

**Table S4** Pairwise genetic distance matrix of *Samsoniella* species for 5P*-TEF* sequences.

| Taxon | 1 | 2 | 3 | 4 | 5 | 6 | 7 | 8 | 9 | 10 | 11 |
| --- | --- | --- | --- | --- | --- | --- | --- | --- | --- | --- | --- |
| 1 (*Samsoniella alpina* YFCC 5818) |  |  |  |  |  |  |  |  |  |  |  |
| 2 (*Samsoniella antleroides* YFCC 6113) | 0.024 |  |  |  |  |  |  |  |  |  |  |
| 3 (*Samsoniella asiatica* YFCC 870 ) | 0.030 | 0.028 |  |  |  |  |  |  |  |  |  |
| 4 (*Samsoniella aurantia* YFCC 874) | 0.027 | 0.014 | 0.036 |  |  |  |  |  |  |  |  |
| 5 (*Samsoniella cardinalis* YFCC 6144) | 0.007 | 0.020 | 0.025 | 0.023 |  |  |  |  |  |  |  |
| 6 (*Samsoniella cristata* YFCC 7004) | 0.027 | 0.003 | 0.031 | 0.017 | 0.023 |  |  |  |  |  |  |
| 7 (*Samsoniella hepiali* ICMM 82-2) | 0.020 | 0.027 | 0.027 | 0.030 | 0.015 | 0.030 |  |  |  |  |  |
| 8 (*Samsoniella lanmaoa* YFCC 6148) | 0.025 | 0.001 | 0.027 | 0.015 | 0.021 | 0.004 | 0.025 |  |  |  |  |
| 9 (*Samsoniella sapaensis* YFCC 873) | 0.017 | 0.012 | 0.024 | 0.012 | 0.013 | 0.015 | 0.020 | 0.014 |  |  |  |
| 10 (*Samsoniella tortricidae* YFCC 6131) | 0.025 | 0.001 | 0.027 | 0.015 | 0.021 | 0.004 | 0.025 | 0.000 | 0.014 |  |  |
| 11 (*Samsoniella yunnanensis* YFCC 1527) | 0.030 | 0.034 | 0.024 | 0.037 | 0.025 | 0.036 | 0.024 | 0.033 | 0.027 | 0.033 |  |

The mean threshold (p-distances) is 0.021.

**Table S5** Pairwise genetic distance matrix of *Samsoniella* species for *RPB1* sequences.

| Taxon | 1 | 2 | 3 | 4 | 5 | 6 | 7 | 8 | 9 | 10 | 11 |
| --- | --- | --- | --- | --- | --- | --- | --- | --- | --- | --- | --- |
| 1 (*Samsoniella alpina* YFCC 5818) |  |  |  |  |  |  |  |  |  |  |  |
| 2 (*Samsoniella antleroides* YFCC 6113) | 0.019 |  |  |  |  |  |  |  |  |  |  |
| 3 (*Samsoniella asiatica* YFCC 870 ) | 0.018 | 0.013 |  |  |  |  |  |  |  |  |  |
| 4 (*Samsoniella aurantia* YFCC 874) | 0.012 | 0.010 | 0.012 |  |  |  |  |  |  |  |  |
| 5 (*Samsoniella cardinalis* YFCC 6144) | 0.001 | 0.021 | 0.019 | 0.013 |  |  |  |  |  |  |  |
| 6 (*Samsoniella cristata* YFCC 7004) | 0.021 | 0.001 | 0.015 | 0.012 | 0.022 |  |  |  |  |  |  |
| 7 (*Samsoniella hepiali* ICMM 82-2) | 0.000 | 0.019 | 0.018 | 0.012 | 0.001 | 0.021 |  |  |  |  |  |
| 8 (*Samsoniella lanmaoa* YFCC 6148) | 0.022 | 0.003 | 0.016 | 0.013 | 0.024 | 0.004 | 0.022 |  |  |  |  |
| 9 (*Samsoniella sapaensis* YFCC 873) | 0.018 | 0.016 | 0.018 | 0.006 | 0.019 | 0.018 | 0.018 | 0.019 |  |  |  |
| 10 (*Samsoniella tortricidae* YFCC 6131) | 0.019 | 0.000 | 0.013 | 0.010 | 0.021 | 0.001 | 0.019 | 0.003 | 0.016 |  |  |
| 11 (*Samsoniella yunnanensis* YFCC 1527) | 0.018 | 0.013 | 0.003 | 0.012 | 0.019 | 0.015 | 0.018 | 0.016 | 0.018 | 0.013 |  |

The mean threshold (p-distances) is 0.014.

**Table S6** Pairwise genetic distance matrix of *Samsoniella* species for *RPB2* sequences.

| Taxon | 1 | 2 | 3 | 4 | 5 | 6 | 7 | 8 | 9 | 10 | 11 |
| --- | --- | --- | --- | --- | --- | --- | --- | --- | --- | --- | --- |
| 1 (*Samsoniella alpina* YFCC 5818) |  |  |  |  |  |  |  |  |  |  |  |
| 2 (*Samsoniella antleroides* YFCC 6113) | 0.004 |  |  |  |  |  |  |  |  |  |  |
| 3 (*Samsoniella asiatica* YFCC 870 ) | 0.010 | 0.010 |  |  |  |  |  |  |  |  |  |
| 4 (*Samsoniella aurantia* YFCC 874) | 0.004 | 0.004 | 0.005 |  |  |  |  |  |  |  |  |
| 5 (*Samsoniella cardinalis* YFCC 6144) | 0.003 | 0.003 | 0.006 | 0.003 |  |  |  |  |  |  |  |
| 6 (*Samsoniella cristata* YFCC 7004) | 0.006 | 0.004 | 0.005 | 0.002 | 0.005 |  |  |  |  |  |  |
| 7 (*Samsoniella hepiali* ICMM 82-2) | 0.003 | 0.003 | 0.006 | 0.003 | 0.000 | 0.005 |  |  |  |  |  |
| 8 (*Samsoniella lanmaoa* YFCC 6148) | 0.006 | 0.004 | 0.005 | 0.002 | 0.005 | 0.000 | 0.005 |  |  |  |  |
| 9 (*Samsoniella sapaensis* YFCC 873) | 0.005 | 0.005 | 0.006 | 0.001 | 0.004 | 0.003 | 0.004 | 0.003 |  |  |  |
| 10 (*Samsoniella tortricidae* YFCC 6131) | 0.006 | 0.004 | 0.005 | 0.002 | 0.005 | 0.000 | 0.005 | 0.000 | 0.003 |  |  |
| 11 (*Samsoniella yunnanensis* YFCC 1527) | 0.007 | 0.007 | 0.002 | 0.003 | 0.006 | 0.003 | 0.006 | 0.003 | 0.004 | 0.003 |  |

The mean threshold (p-distances) is 0.004.

**Table S7** Pairwise genetic distance matrix of *Samsoniella* species for *ACT* sequences.

| Taxon | 1 | 2 | 3 | 4 | 5 | 6 | 7 | 8 | 9 | 10 | 11 |
| --- | --- | --- | --- | --- | --- | --- | --- | --- | --- | --- | --- |
| 1 (*Samsoniella alpina* YFCC 5818) |  |  |  |  |  |  |  |  |  |  |  |
| 2 (*Samsoniella antleroides* YFCC 6113) | 0.008 |  |  |  |  |  |  |  |  |  |  |
| 3 (*Samsoniella asiatica* YFCC 870 ) | 0.008 | 0.005 |  |  |  |  |  |  |  |  |  |
| 4 (*Samsoniella aurantia* YFCC 874) | 0.013 | 0.007 | 0.010 |  |  |  |  |  |  |  |  |
| 5 (*Samsoniella cardinalis* YFCC 6144) | 0.001 | 0.010 | 0.007 | 0.014 |  |  |  |  |  |  |  |
| 6 (*Samsoniella cristata* YFCC 7004) | 0.014 | 0.006 | 0.008 | 0.013 | 0.013 |  |  |  |  |  |  |
| 7 (*Samsoniella hepiali* ICMM 82-2) | 0.006 | 0.010 | 0.010 | 0.014 | 0.005 | 0.013 |  |  |  |  |  |
| 8 (*Samsoniella lanmaoa* YFCC 6148) | 0.010 | 0.001 | 0.006 | 0.008 | 0.011 | 0.005 | 0.011 |  |  |  |  |
| 9 (*Samsoniella sapaensis* YFCC 873) | 0.014 | 0.008 | 0.008 | 0.001 | 0.013 | 0.012 | 0.013 | 0.010 |  |  |  |
| 10 (*Samsoniella tortricidae* YFCC 6131) | 0.010 | 0.001 | 0.004 | 0.008 | 0.008 | 0.005 | 0.008 | 0.002 | 0.007 |  |  |
| 11 (*Samsoniella yunnanensis* YFCC 1527) | 0.010 | 0.006 | 0.001 | 0.011 | 0.008 | 0.010 | 0.011 | 0.007 | 0.010 | 0.005 |  |

The mean threshold (p-distances) is 0.008.

**Table S8** Pairwise genetic distance matrix of *Samsoniella* species for *TUB* sequences.

| Taxon | 1 | 2 | 3 | 4 | 5 | 6 | 7 | 8 | 9 | 10 | 11 |
| --- | --- | --- | --- | --- | --- | --- | --- | --- | --- | --- | --- |
| 1 (*Samsoniella alpina* YFCC 5818) |  |  |  |  |  |  |  |  |  |  |  |
| 2 (*Samsoniella antleroides* YFCC 6113) | 0.011 |  |  |  |  |  |  |  |  |  |  |
| 3 (*Samsoniella asiatica* YFCC 870 ) | 0.009 | 0.016 |  |  |  |  |  |  |  |  |  |
| 4 (*Samsoniella aurantia* YFCC 874) | 0.004 | 0.011 | 0.009 |  |  |  |  |  |  |  |  |
| 5 (*Samsoniella cardinalis* YFCC 6144) | 0.002 | 0.009 | 0.007 | 0.002 |  |  |  |  |  |  |  |
| 6 (*Samsoniella cristata* YFCC 7004) | 0.004 | 0.007 | 0.009 | 0.004 | 0.002 |  |  |  |  |  |  |
| 7 (*Samsoniella hepiali* ICMM 82-2) | 0.003 | 0.010 | 0.008 | 0.003 | 0.001 | 0.003 |  |  |  |  |  |
| 8 (*Samsoniella lanmaoa* YFCC 6148) | 0.009 | 0.004 | 0.014 | 0.009 | 0.007 | 0.004 | 0.008 |  |  |  |  |
| 9 (*Samsoniella sapaensis* YFCC 873) | 0.004 | 0.011 | 0.009 | 0.002 | 0.002 | 0.004 | 0.003 | 0.009 |  |  |  |
| 10 (*Samsoniella tortricidae* YFCC 6131) | 0.010 | 0.003 | 0.015 | 0.010 | 0.008 | 0.008 | 0.009 | 0.008 | 0.010 |  |  |
| 11 (*Samsoniella yunnanensis* YFCC 1527) | 0.009 | 0.016 | 0.002 | 0.009 | 0.007 | 0.009 | 0.008 | 0.014 | 0.009 | 0.015 |  |

The mean threshold (p-distances) is 0.007.

**Table S9** Pairwise genetic distance matrix of *Samsoniella* species for *MCM7* sequences.

| Taxon | 1 | 2 | 3 | 4 | 5 | 6 | 7 | 8 | 9 | 10 | 11 |
| --- | --- | --- | --- | --- | --- | --- | --- | --- | --- | --- | --- |
| 1 (*Samsoniella alpina* YFCC 5818) |  |  |  |  |  |  |  |  |  |  |  |
| 2 (*Samsoniella antleroides* YFCC 6113) | 0.018 |  |  |  |  |  |  |  |  |  |  |
| 3 (*Samsoniella asiatica* YFCC 870 ) | 0.018 | 0.016 |  |  |  |  |  |  |  |  |  |
| 4 (*Samsoniella aurantia* YFCC 874) | 0.028 | 0.020 | 0.027 |  |  |  |  |  |  |  |  |
| 5 (*Samsoniella cardinalis* YFCC 6144) | 0.002 | 0.016 | 0.016 | 0.027 |  |  |  |  |  |  |  |
| 6 (*Samsoniella cristata* YFCC 7004) | 0.020 | 0.008 | 0.018 | 0.018 | 0.018 |  |  |  |  |  |  |
| 7 (*Samsoniella hepiali* ICMM 82-2) | 0.003 | 0.015 | 0.015 | 0.025 | 0.002 | 0.016 |  |  |  |  |  |
| 8 (*Samsoniella lanmaoa* YFCC 6148) | 0.021 | 0.006 | 0.016 | 0.023 | 0.020 | 0.011 | 0.018 |  |  |  |  |
| 9 (*Samsoniella sapaensis* YFCC 873) | 0.030 | 0.021 | 0.028 | 0.008 | 0.028 | 0.020 | 0.027 | 0.025 |  |  |  |
| 10 (*Samsoniella tortricidae* YFCC 6131) | 0.015 | 0.010 | 0.013 | 0.016 | 0.013 | 0.008 | 0.011 | 0.013 | 0.018 |  |  |
| 11 (*Samsoniella yunnanensis* YFCC 1527) | 0.016 | 0.015 | 0.002 | 0.025 | 0.015 | 0.016 | 0.013 | 0.015 | 0.027 | 0.011 |  |

The mean threshold (p-distances) is 0.017.
