# Supplementary material for: ﻿Phylogeny and species delimitations in the economically, medically, and ecologically important genus Samsoniella (Cordycipitaceae, Hypocreales)
Source: MycoKeys. 2023 Oct 3;99:227–50. doi: 10.3897/mycokeys.99.106474 (PMC10565569; doi:10.3897/mycokeys.99.106474)
Supplement: Supplementary material 3 — Phylogenetic tree of Samsoniella [file mycokeys-99-227-s003.docx]

**
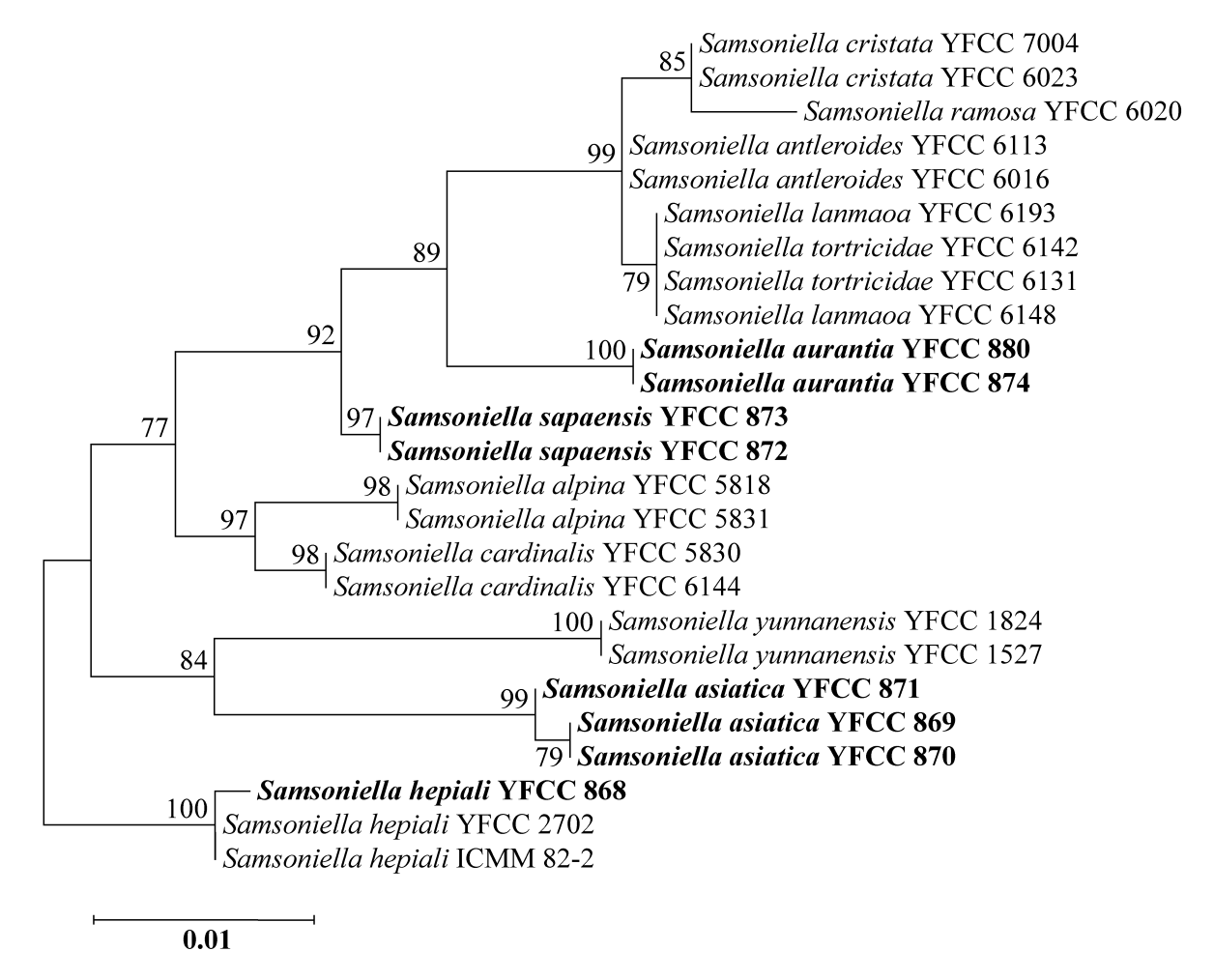
**

**Figure S1.** Phylogenetic tree of *Samsoniella* based on Maximum Likelihood (ML) analysis from the 5P*-TEF* sequences. Statistical support values (≥50%) are shown at the nodes for ML boostrap support.


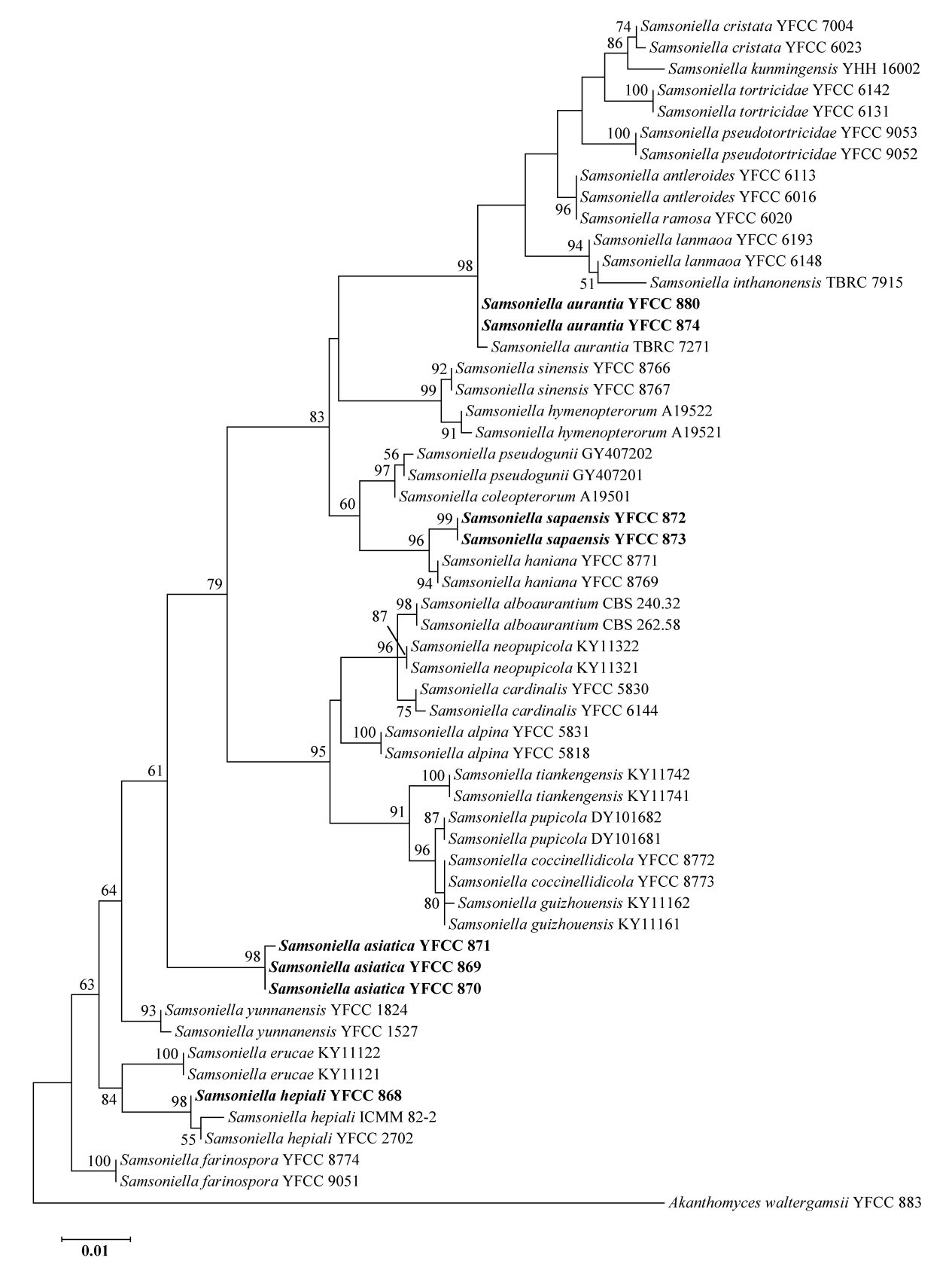


**Figure S2.** Phylogenetic tree of *Samsoniella* based on Maximum Likelihood (ML) analysis from the 3P*-TEF* sequences. Statistical support values (≥50%) are shown at the nodes for ML boostrap support.


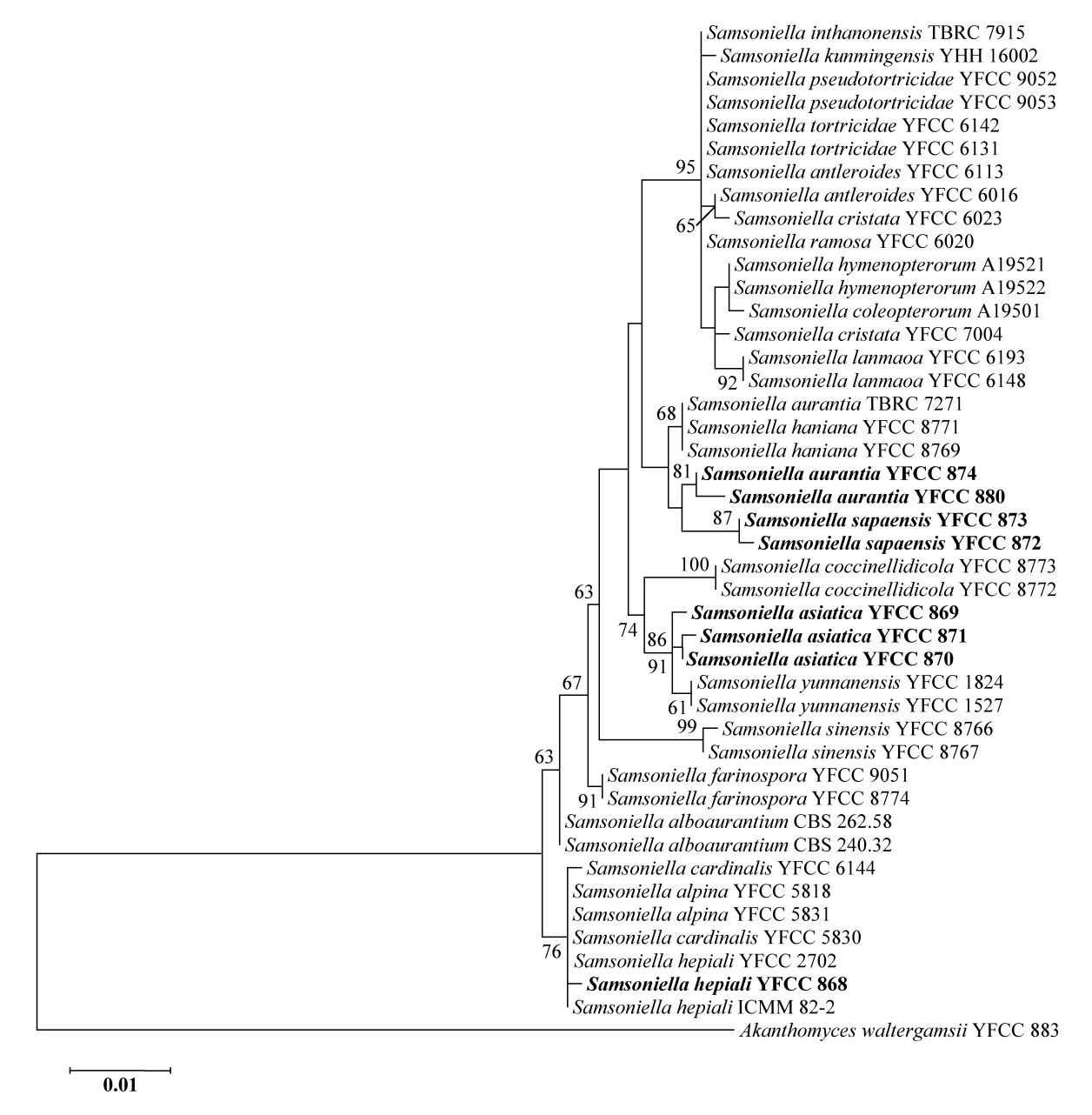


**Figure S3.** Phylogenetic tree of *Samsoniella* based on Maximum Likelihood (ML) analysis from the *RPB1* sequences. Statistical support values (≥50%) are shown at the nodes for ML boostrap support.


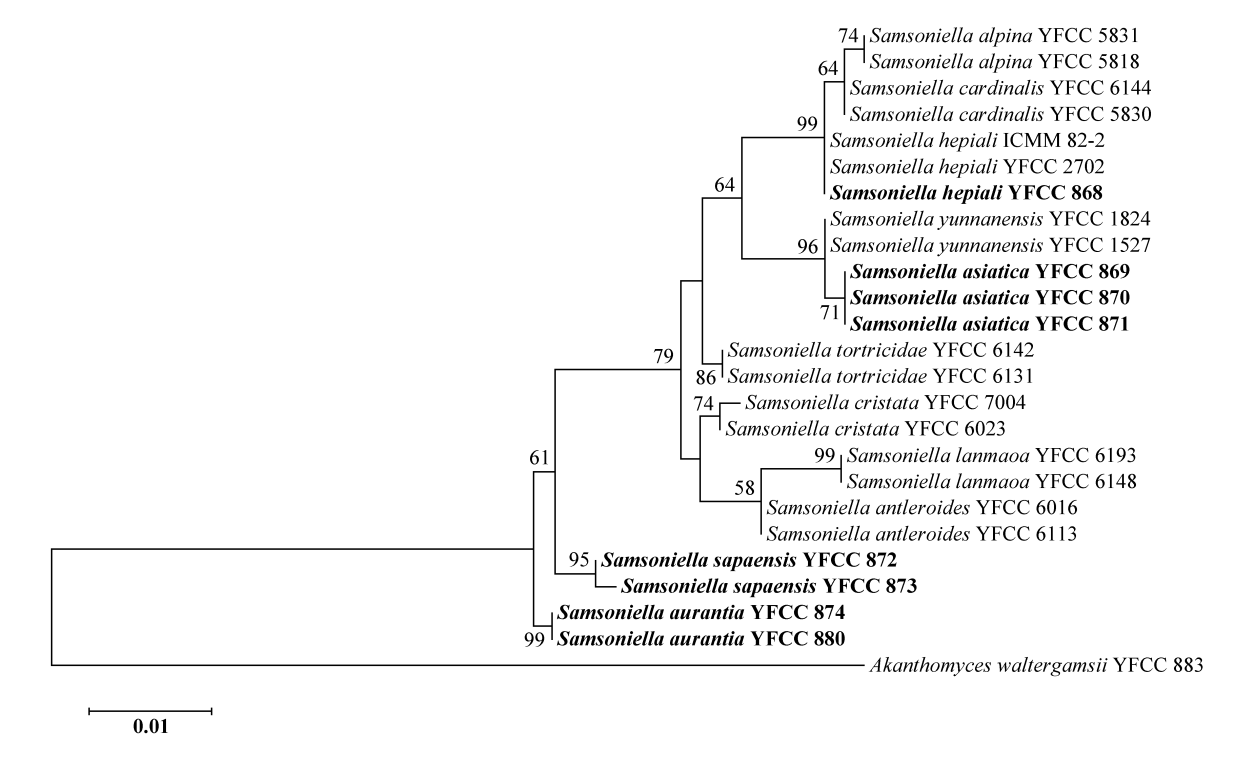


**Figure S4.** Phylogenetic tree of *Samsoniella* based on Maximum Likelihood (ML) analysis from the *MCM7* sequences. Statistical support values (≥50%) are shown at the nodes for ML boostrap support.
