# Supplementary material for: ﻿Phylogeny and species delimitations in the economically, medically, and ecologically important genus Samsoniella (Cordycipitaceae, Hypocreales)
Source: MycoKeys. 2023 Oct 3;99:227–50. doi: 10.3897/mycokeys.99.106474 (PMC10565569; doi:10.3897/mycokeys.99.106474)
Supplement: Supplementary material 4 — Highlights [file mycokeys-99-227-s004.docx]

Highlights

- Our results re-established well-supported boundaries in the genus *Samsoniella* and allowed for the delimitation of 26 species in this genus.
- Two new *Samsoniella* species, *S. asiatica* and *S. sapaensis*, were proposed.
- In this study, anamorphically typified *S. aurantia* and *S. hepiali* were grown for the first time from teleomorph stromata.
- The genetic divergence comparisons showed that the ITS, *RPB2*, *ACT*, and *TUB* sequences provided little valuable information with which to separate *Samsoniella* spp. In contrast, sequence data for 3P_*TEF*, 5P_*TEF*, *RPB1*, and *MCM7* provided good resolution of *Samsoniella* species.
